# Synthesis, Spectroscopy, Electrochemistry and DFT of Electron-Rich Ferrocenylsubphthalocyanines

**DOI:** 10.3390/molecules25112575

**Published:** 2020-06-01

**Authors:** Pieter J. Swarts, Jeanet Conradie

**Affiliations:** 1 Department of Chemistry, University of the Free State, Bloemfontein 9300, South Africa; swarts.pieter@gmail.com; 2 Department of Chemistry, UiT—The Arctic University of Norway, N-9037 Tromsø, Norway

**Keywords:** subphthalocyanines, ferrocene, redox potentials, DFT, electron rich

## Abstract

A series of novel ferrocenylsubphthalocyanine dyads Y-BSubPc(H)_12_ with ferrocenyl-carboxylic acids Y-H = (FcCH_2_CO_2_-H), (Fc(CH_2_)_3_CO_2_-H) or (FcCO(CH_2_)_2_CO_2_-H) in the axial position were synthesized from the parent Cl-BSubPc(H)_12_ via an activated triflate-SubPc intermediate. UV/Vis data revealed that the axial ferrocenyl-containing ligand did not influence the Q-band maxima compared to Cl-BSubPc(H)_12_. A combined electrochemical and density functional theory (DFT) study showed that Fe group of the ferrocenyl-containing axial ligand is involved in the first reversible oxidation process, followed by a second oxidation localized on the macrocycle of the subphthalocyanine. Both observed reductions were ring-based. It was found that the novel Fc(CH_2_)_3_CO_2_BSubPc(H)_12_ exhibited the lowest first macrocycle-based reduction potential (−1.871 V vs. Fc/Fc^+^) reported for SubPcs till date. The oxidation and reduction values of Fc(CH_2_)_n_CO_2_BSubPc(H)_12_ (*n* = 0–3), FcCO(CH_2_)_2_CO_2_BSubPc(H)_12_, and Cl-BSubPc(H)_12_ illustrated the electronic influence of the carboxyl group, the different alkyl chains and the ferrocenyl group in the axial ligand on the ring-based oxidation and reduction values of the SubPcs.

## 1. Introduction

**Figure 1:**
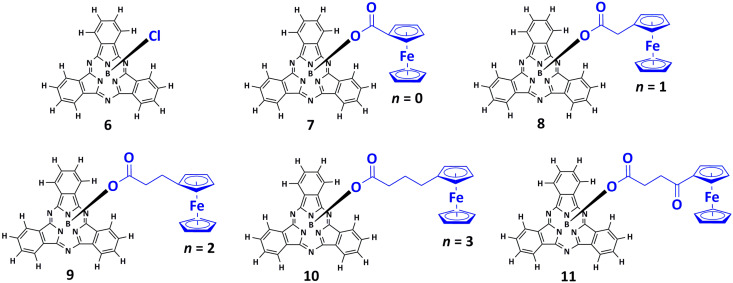
Structures of ClBSubPc(H)_12_, **6**, FcCO_2_BSubPc(H)_12_, **7** [21], FcCH_2_CO_2_BSubPc(H)_12_, **8** (novel), Fc(CH_2_)_2_CO_2_BSubPc(H)_12_, **9** [24], Fc(CH_2_)_3_CO_2_BSubPc(H)_12_, **10** (novel) and FcCO(CH_2_)_2_CO_2_BSubPc(H)_12_, **11** (novel), containing Cl or the ferrocenylcarboxylic acids FcCO_2_H (**1**), FcCH_2_CO_2_H (**2**), Fc(CH_2_)_2_CO_2_H (**3**), Fc(CH_2_)_3_CO_2_H (**4**) and FcCO(CH_2_)_2_CO_2_H (**5**) in the axial position. *n* = number of alkyl groups in the axial ligand.

The discovery of ferrocene in 1951 [1] unlocked an entirely new research field and over the years ferrocene has been extensively studied and well-reviewed in organic and inorganic chemistry [2,3,4,5]. The research of ferrocene-containing compounds thrives, and new research keeps on growing due to their varying successful applications. This includes asymmetric catalysis [6,7], non-linear optics [8], antineoplastic properties [9,10], antimalarial activity [11] and especially electrochemistry due to the ideal redox behavior of the Fe^II/III^ couple [6,9,12]. For a series of four ferrocenyl carboxylic acid dyads Fc(CH_2_)_n_CO_2_H with *n* = 0 (**1**), 1 (**2**), 2 (**3**) or 3 (**4**) (Figure 1), it was found that as the length of the alkyl chain separating the ferrocene moiety and electron-withdrawing carboxy group decreases, the formal reduction potential of Fe of the ferrocenyl group also decreases [13,14]. The electron-withdrawing carboxyl group directly bound to ferrocene in **1** and FcCO(CH_2_)_2_CO_2_H (**5**), led to an increase in the formal reduction potential of the ferrocene moiety compared to free ferrocene [13,14].

Subphthalocyanines such as ClBSubPc(H)_12_, **6**, have been used in research for almost 50 years [15] due to their diverse applications including light-emitting diodes [16], dye-sensitized solar cells [17], sensors [18] and photodynamic therapy [19]. The uses and reactivity of SubPcs can be modified by substituting the axial ligand as well as by functionalizing the ring substituents [18]. Redox data of the ring-based oxidation and reduction processes of SubPcs showed that axial or peripheral substitution has a smaller influence on the shift of the oxidation and reduction potential of the SubPc (ca 0.1 V shift) than non-peripheral substitution (ca 0.3 V shift) [20]. Ferrocenylsubphthalocyanine dyads with a direct ferrocene-boron or substituted ferrocene-boron bond have been investigated by Nemykin and co-workers [21,22]. They found that the first oxidation process in these ferrocenylsubphthalocyanine dyads is ferrocene based, while second oxidation and the two oberverved reduction processes are centered at the macrocyclic ligand of the SubPc [21,22].

**Scheme 1:**
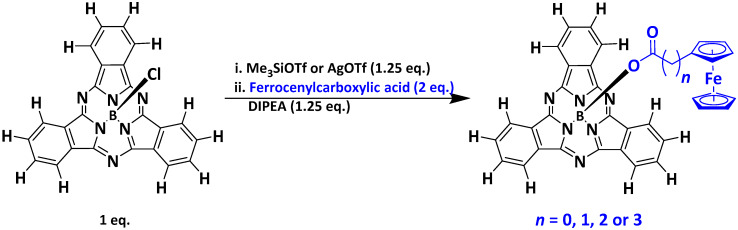
Reaction scheme for the synthesis of FcCO_2_BSubPc(H)_12_, **7,** FcCH_2_CO_2_BSubPc(H)_12_, **8,** Fc(CH_2_)_2_CO_2_BSubPc(H)_12_, **9** [24], and Fc(CH_2_)_3_CO_2_BSubPc(H)_12_, **10**, (similar for FcCO(CH_2_)_2_CO_2_BSubPc(H)_12_, **11**) from ClBSubPc(H)_12_, **6**. Note: AgOTf = silver trifluoromethanesulfonate, Me_3_SiOTf = Trimethylsilyl trifluoromethanesulfonate and DIPEA = *N*,*N*-Diisopropylethylamine.

In this paper, we report the synthesis, characterisation and electrochemical studies of three novel ferrocenylsubphthalocyanine dyads **8**, **10** and **11**. The appropriate ferrocenyl carboxylic acids **2**, **4** and **5** was directly bound to the boron atom in the axial position of Cl-BSubPc(H)_12_, **6**, to form the three new ferrocenylsubphthalocyanine dyads, FcCH_2_CO_2_BSubPc(H)_12_, **8**, Fc(CH_2_)_3_CO_2_BSubPc(H)_12_, **10**, and FcCO(CH_2_)_2_CO_2_BSubPc(H)_12_, **11** (see Scheme 1 [23] and Figure 1). For comparative purposes, the two known ferrocenylsubphthalocyanine dyads **7** [21] and **9** [24] were included in this study to systematically evaluate (i) the effect of the electron rich macrocycle of SubPcs **7**–**11** on the formal reduction potential of Fe of the ferrocenyl group of the ferrocenylcarboxylic acid moieties of **7**–**11**, and (ii) the influence of the axially ferrocenylcarboxylic acid **1**–**5** on the UV/vis maxima and the ring-based oxidations and reductions of the ferrocenylsubphthalocyanine dyads **7**–**11**.

## 2. Results and Discussion

### 2.1. Synthesis

The free ferrocenylcarboxylic acids **1**–**5**, were synthesized using slightly modified methods than previously published [13], as described in our previous publication [14]. The synthesis of ferrocenylsubphthalocyanine dyads **7**, **8**, **10** and **11** was complex due to the moisture sensitivity of the reactions. In the first step any of the two well-known halophiles, such as Me_3_Si groups or Ag^+^ ions are used to irreversibly substitute the axial chloride in the axial position [23]. We found that using Me_3_SiOTf did not give desirable yields. Using AgOTf, however, enabled us to increase our yields by more than 30% compared to previous studies [21]. Once the activated triflate species was formed the activated SubPc showed considerable reactivity toward the different ferrocenyl acids **1**, **2**, **4** and **5** see Scheme 1. The success of the synthesis is drastically affected when not working under strict Schlenk conditions. SubPcs **7**, **8**, **10** and **11** were soluble in common organic solvents such as dichloromethane (DCM), chloroform and tetrahydrofuran (THF).

### 2.2. ^1^H NMR

The ^1^H-NMR results showed that signals of the SubPc ring protons (H) shifted upfield by ca. 0.04–0.07 ppm for SubPcs **7**–**11** relative to the signals of the parent ring protons ClBSubPc(H)_12_, **6**. The most significant effect on ^1^H-NMR was observed for the signals of the ferrocenyl axial ligands peaks. Ferrocenyl of SubPc **7** (signals of the protons of substituted-Cp = 3.96 and 3.95, unsubstituted-Cp = 3.63) is the closest to the electron rich macrocycle of SubPc and as a result the signals of the protons of the substituted and unsubstituted-Cp rings shift the furthest upfield with ppm shifts between 0.50 and 0.88 ppm compared to ferrocenyl acid **1** (substituted-Cp = 4.84 and 4.45, unsubstituted-Cp = 4.24). With the increase of (CH_2_) linker groups the distance between the ferrocenyl moiety and SubPc increases and as a result the ferrocene peaks shift less upfield as the chain lengths increase.

### 2.3. UV/vis

**Figure 2:**
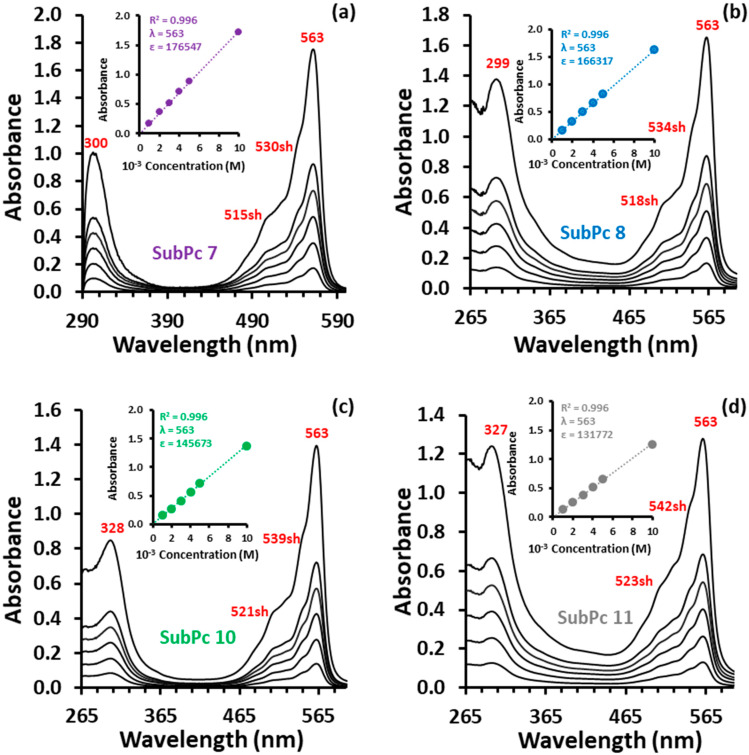
(**a**)–(**d**) The UV/vis spectra of SubPc (**7**, **8**, **10** and **11**) at concentrations 0.01, 0.02, 0.03, 0.04, 0.05 and 0.10 (× 10^−3^ M), obtained with a 1 cm pathlength cuvette with tetrahydrofuran (THF) as solvent. UV/vis spectra of SubPc **9** can be found in reference [24]. Insert: The Beer-Lambert correlation between the absorbance A and concentration of FcCO_2_BSubPc(H)_12_, **7** (ε = 176547 dm^3^ mol^−1^ cm^−1^); FcCH_2_CO_2_BSubPc(H)_12_, **8** (ε = 166317 dm^3^ mol^−1^ cm^−1^), Fc(CH_2_)_2_CO_2_BSubPc(H)_12_, **9** (ε = 153630 dm^3^ mol^−1^ cm^−1^ [24]), Fc(CH_2_)_3_CO_2_BSubPc(H)_12_, **10** (ε = 145673 dm^3^ mol^−1^ cm^−1^) and FcCO(CH_2_)_2_CO_2_BSubPc(H)_12_, **11** (ε = 131772 dm^3^ mol^−1^ cm^−1^) at indicated wavelength in nm.

As usually found for SubPcs, the UV/Vis spectra of SubPcs **7**–**11** exhibited the two main transitions, the Soret band between 250 and 350 nm, and the Q-band between 450 and 620 nm, see Figure 2. The substitution of the parent macrocycles axial chloride with the different ferrocenylcarboxylic acid groups, had a negligible effect on the Q-bands position, see Table 1. In a similar fashion there was no shift when comparing the Q-bands of SubPcs **7**–**11** to **6**. The similar Soret and Q-bands for **6** and SubPcs **7**–**11** indicates, in agreement with density functional theory (DFT) and time-dependent density-functional theory (TDDFT) calculations (see discussion in Section 2.5 below), that the Soret and Q-bands involve π-π* transitions. SubPcs **7** to **11** followed the Beer-Lambert law and no aggregation in the concentration range of 0.01–0.10 (× 10^−3^ M) was observed, see Figure 2.

### 2.4. Cyclic Voltammetry

**Scheme 2:**
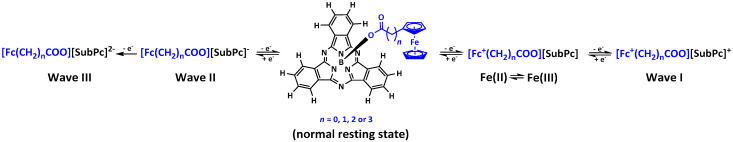
The reaction scheme of the redox signals of SubPcs **7**–**10** in dichloromethane (DCM) as solvent, see Figure 3. Reaction scheme for **11** is similar.

**Figure 3:**
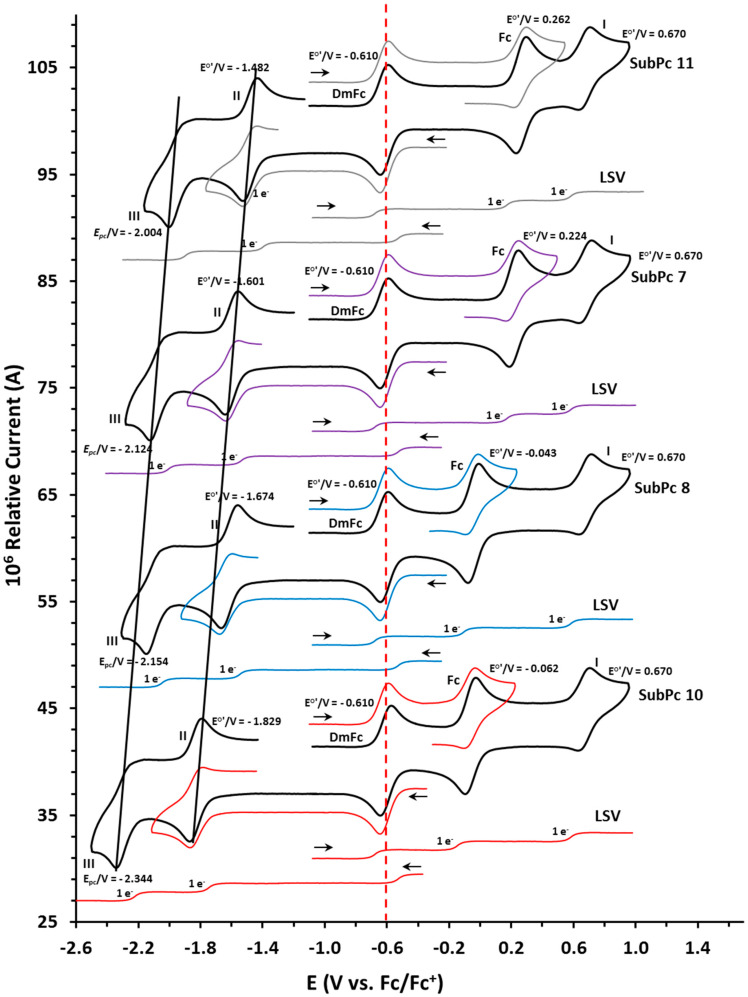
Cyclic voltammetry (CVs) and linear sweep voltammetry (LSVs) of SubPc, **7**—(purple), **8**—(blue), **10**—(red) and **11**—(grey) in dichloromethane (DCM). Concentration of **7, 8, 10** and **11** = 0.0005 mol dm^−3^. Scan rate for CVs is 0.100 V s^−1^ and LSVs at 0.001 V s^−1^. Scan directions are indicated at starting point of each scan. DmFc was used as internal reference with *E*°’ (DmFc) = −0.610 vs. free Fc/Fc^+^ at 0 V.

The redox properties of the ferrocenylsubphthalocyanine dyads, SubPc **7**, **8**, **10** and **11** were examined utilizing cyclic voltammetry (CVs) and linear sweep voltammetry (LSV). The CVs and the LSVs of SubPc **7**, **8**, **10** and **11**, performed at 25 °C in dichloromethane (DCM) at a scan rate of 0.100 Vs^−1^ are shown in Figure 3 with the relevant electrochemical data summarised in Table 2. Data of the free ferrocenylcarboxylic acids **1**–**5** [14] and SubPcs **6** [20] and **9** [24] are added for comparative reasons in Table 2. The CVs of SubPc **7**, **8**, **10** and **11** (Figure 3) showed two oxidation and two reduction peaks in the experimental solvent window of DCM. The oxidation of the Fe group (Fe^II/III^) of the ferrocenyl moiety on the axial ligand is the first observed redox process for all five SubPc **7**–**11**. The assignment that the Fe group is oxidised first, is supported by DFT calculations (see computational analysis below) and is in agreement with literature [21,22,24]. Both oxidation and the first reduction peaks are chemically reversible with peak ratios approaching 1 and peak current separations, Δ*E*_p_, of 0.074–0.076 (ferrocenyl oxidation), 0.080–0.084 V (wave I in Figure 3) and 0.082–0.086 V (wave II in Figure 3). The second ring-based reduction (wave III in Figure 3) were irreversible and did not show any re-oxidation peaks. The linear sweep voltammetry showed, as expected, 1 e^−^ redox couples for Fc and waves I to III. The reaction scheme for the redox signals of SubPc **7**–**10** (similar for **11**) is given in Scheme 2. The electrochemical band gaps estimated from the SubPc-based redox potentials (peaks I and II) become wider in the order of compound **11**, **7**, **8**, **9** and **10**. However, the first ring-based oxidation process (peak I) originates from a cationic SubPc and not from the neutral SubPc as is the case of the Q-band maximum, that is constant for SubPcs **7**–**11**.

#### 2.4.1. Effect of SubPc on Ferrocenyl Moiety Oxidation

The oxidation potentials, *E*°’, of the Fe group (Fe^II/III^) of the ferrocenyl moiety on the axial ligand of SubPcs **7**–**11** range between −0.062 and 0.262 V and with Δ*E_p_* between 0.074 and 0.076 V (Figure 3 and Table 2). Comparing *E*°’ (of the first process) of the Fc moiety of SubPcs **7**–**11** with the *E*°’ of the free ferrocenyl acids **1**–**5** (obtained under the experimental same conditions [14] as the SubPcs **7**–**11**) it is clear that the aromatic SubPc ring acts as an electron-donating specie in the complex, decreasing (lower oxidation potential) *E*°’ of the Fe group of SubPcs **7**–**11** with 0.060–0.038 V relative to *E*°’ of the Fe group of the free ferrocenylcarboxylic acids **1**–**5**. The largest electron-donating effect was, as expected, on the ferrocenyl moiety closest to the ring, with a decrease of 0.060 V between **7** (0.224 V) and **1** (0.284 V). As the chain length increases with (CH_2_) groups, the effect of the SubPc on ferrocenyl oxidation becomes less. The Fe group in SubPc **10** with three (CH_2_) groups separating the ferrocenyl moiety from the SubPc, is more shielded from the electron-donating effect of the SubPc and as a result *E*°’ of Fe only shifts with only 0.038 V between **10** (−0.062 V) and **4** (−0.024 V). With an additional carbonyl group as well as the (CH_2_) groups in SubPc **11**, *E*°’ of the Fe group of SubPc **11** shifted the least in the range namely, with only a 0.033 V between **11** (0.262 V) and **5** (0.295 V).

#### 2.4.2. Effect of Chain Length on Ferrocenyl Moiety Oxidation

**Figure 4:**
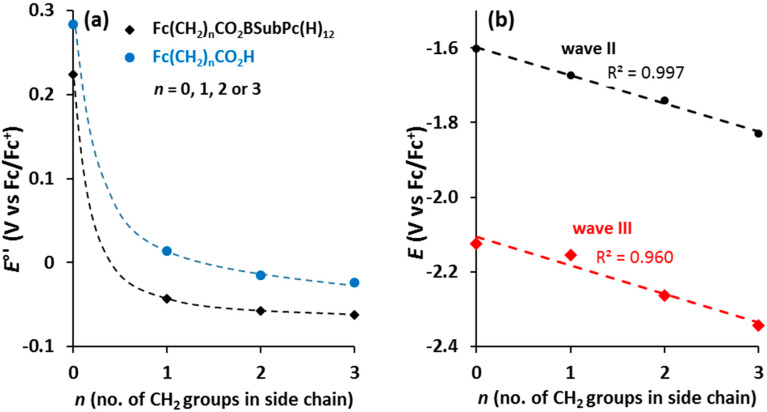
(**a**) Relationship between *E*°’ of the Fe group and the number *n* of CH_2_ groups in the side chain of Fc–(CH_2_)_n_–CO_2_ for ferrocenyl carboxylic acids **1**–**4** and ferrocenylsubphthalocyanine dyads **7**–**10**. Data of **1** – **4** from reference [14], data of **9** from reference [24]. (**b**) Relationship between *E*°’(wave II) and *E*_pc_ (wave III) and *n*, of the first and second ring-based reductions respectively, of ferrocenylsubphthalocyanine dyads **7**–**10**.

The formal reduction potential *E*°’ of Fe in the axial ligand of SubPc **7** is at 0.224 V compared to free ferrocene 0 V. With one additional CH_2_ spacer group between ferrocene and the SubPc, *E*°’ of Fe is significantly shifted by 0.267 V to −0.043 V in SubPc **8**. SubPcs **9** and **10** had two and three additional CH_2_ spacer groups respectively, with *E*°’ of the ferrocene group decreasing to −0.058 V and −0.062 V respectively. There was an exponential decrease to lower oxidation potential with an increase in (CH_2_)_n_ chain lengths for SubPc **7**–**10**, see Figure 4a. This is because Fe of the ferrocene group is increasingly shielded from the electron donating effect of the SubPc and electron-withdrawing effect of the carboxy group [14] as the (CH_2_)_n_ chain length increases. This trend was similar as observed for the free ferrocenylcarboxylic acids **1**–**4 [14]**, see Figure 4a.

#### 2.4.3. Effect of Carboxyl and Carbonyl Group on Ferrocenyl Moiety Oxidation

The formal reduction potential *E*°’ of the Fe group of SubPc **7**, separated by a carboxyl group from the electron donating SubPc, is at 0.224 V compared to free ferrocene 0 V, implying that the electron withdrawing effect of the carboxyl group is larger than the electron donating effect of the SubPc on the *E*°’ of the Fe group. The additional electron withdrawing carbonyl group bound next to the ferrocenyl moiety in SubPc **11** resulted in the highest oxidation potential of the Fe group with *E*°’ at 0.262 V, Figure 3. With the two electron withdrawing CO groups in SubPc **11** the oxidation potential was 0.038 V higher than *E*°’ of Fe in SubPc **7** (0.224 V) containing only one electron withdrawing CO group.

#### 2.4.4. Ring Based Reductions

The first (wave II) and second (wave III) ring-based reductions of SubPcs **7**–**11** are lower than that of SubPc **6**. The neutral axial ferrocenylcarboxylic ligands of SubPcs **7**–**11** thus have a net electron donating effect on the aromatic ring electrons of SubPcs **7**–**11** compared to Cl in SubPc **6**. The net electron donating effect of the neutral axial ferrocenylcarboxylic ligand is a combination of the electron-withdrawing effect of the carboxyl group [14], the electron donating effect of the alkyl chain [10,14,25] and the electron donating effect of ferrocenyl [26]. Both ring-based reductions of SubPcs **7**–**11**, redox waves II and III in Figure 3 and Table 2, followed the same trend, namely the reduction value decreases near linear as the number of (CH_2_) groups, *n*, in the different axially bonded ferrocenyl carboxylic acid moieties (Fc-(CH_2_)_n_-CO_2_) increases in SubPcs **7**–**10**, with the reduction values of SubPc **11** higher than that of SubPc **7**, see Figure 4b. The aromatic ring electron density of the SubPcs systematically increased as *n* of the alkyl group in –OCO(CH_2_)_n_-Fc increases. SubPc **10** exhibited the lowest first ring-based reduction potential (−1.872 V), reported to date [18,21,22,27], due to the (OOC(CH_2_)_3_)Fc group’s net electron-donating effect. The donating effect is the most prominent on SubPc **10** containing the longest alkyl chain. Propyl (*n* = 3) is more electron donating than ethyl (*n* = 2) that is more electron donating then methyl (*n* = 1) [10,14,25].

#### 2.4.5. Ring Based Oxidation

*E*°’ of the first ring-based oxidation of SubPcs **7**–**11** (wave I in Figure 3) is exactly the same, with *E*°’ = 0.670 V for all five complexes **7**–**11**. This is because the charge located on the ferrocenium group (Fc^+^) in the different oxidised ferrocenyl carboxylic acid moieties (Fc^+^-(CH_2_)_n_-CO_2_) and (Fc^+^-CO(CH_2_)_2_-CO_2_) for SubPcs **7**–**11**, is largely isolated from the rest of the molecule. The Fc^+^ group is highly electronegative [26,28], withdrawing any available electron density from the alkyl groups bonded to it. The first ring-based oxidation of SubPcs **7**–**11** is consequently influenced by the net electron withdrawing effect of the oxidized axial ferrocenylcarboxylic ligands of SubPcs **7**–**11** that is a combination of the electron-withdrawing effect of the carboxyl group [14], the electron donating effect of the alkyl chain [10,14,25] and the highly electron withdrawing effect of ferrocenium [26,28], shifting the first ring-based oxidation of SubPcs **7**–**11** with exactly the same value namely 0.042 V more positive than *E*°’ of the first ring-based oxidation of SubPcs **6** at 0.628 V. The net electron withdrawing effect of the oxidized axial ferrocenylcarboxylic ligands in **7**–**11** is thus larger than the electron withdrawing effect of Cl in **6**, on the aromatic ring electron density of the SubPcs. It was possible to get chemically reversible ring-based oxidation with peak current ratios approaching 1 and peak current separation Δ*E_p_* between 0.080 and 0.084 V, see Table 2.

### 2.5. Computational Analysis

**Figure 5:**
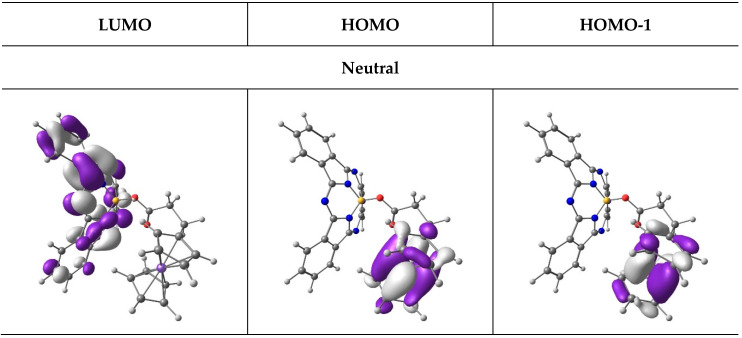

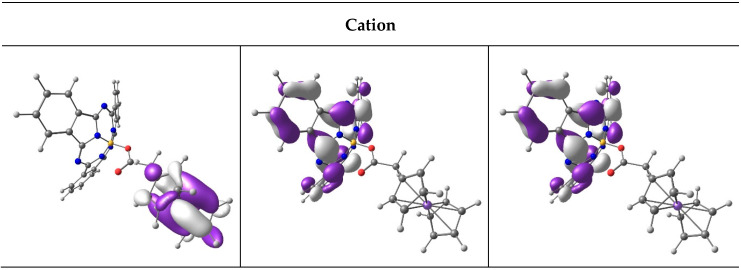
Selected PBE1PBE/6-311G(d,p) frontier molecular orbitals (MOs) for cation SubPc, **8**. A contour of 0.03 e/Å^3^ was used for the orbital plots. Colour code of atoms (online version): Fe (purple), B (yellow), C (grey), O (red), H (white). HOMO = highest occupied molecular orbital and LUMO = lowest unoccupied molecular orbital.

The structure of the ferrocenylcarboxylic-containing SubPc dyads **7**–**11**, were optimised using density functional theory (DFT) methods to solve the Schrödinger equations in order to gain further insight into the redox properties of the ferrocene dyads. In agreement with previous studies on related SubPcs, the top three high-lying occupied molecular orbitals (MOs) of the neutral species are of iron-d character while the lowest unoccupied MO (LUMO) and the LUMO+1 have π-ring character [21,24]. This confirm Fe(II) to Fe(III) oxidation and ring-based reduction respectively. However, since the top three highest occupied MOs (HOMOs) of **7**–**11** are all iron-d based, and Fe(III) to Fe(IV) oxidation is not expected, it was essential to also optimise the cation (oxidised) species, to locate the locus of the second experimentally observed oxidation. It is known that orbitals can rearrange upon oxidation [29,30,31]. The DFT results of oxidised SubPcs **7**–**11** all showed that the LUMO is of iron-d character (the first oxidation, see Figure 5) and the HOMO is on the SubPc ring (the second oxidation, see Figure 5). HOMO-1, also on the SubPc ring, will be the second ring oxidation, however, it is out of the solvent window in cyclic voltammetry scans and not experimentally observed.

For all neutral SubPcs **7**–**11** it was found that the HOMO-3 is located on the macrocycle of the SubPc. The π-π* transition of the Q-band excitation [32] of SubPcs **7**–**11**, in agreement with TDDFT calculations, originates thus from HOMO-3 to LUMO excitation of the neutral SubPc. The DFT calculated energy difference between HOMO-3 and LUMO is the same for SubPcs **7**–**11** within 0.003 eV, explaining why the experimental Q-band maxima of SubPcs **7**–**11** is exactly the same, namely 563 nm.

## 3. Materials and Methods

Solid reagents (Sigma-Aldrich, Johannesburg, South Africa, STREM, Newburyport, MA, USA and Merck, Johannesburg, South Africa) were used as received. Liquid reagents (Sigma-Aldrich and Merck) were used without any further purification unless specified otherwise. Solvents were distilled, and water was double distilled. Organic solvents used in this study were dried according to published methods [33]. Melting points are uncorrected and were determined with a BX 51 microscope (Olympus, Johannesburg, South Africa) equipped with a THMS 600 hot stage (Linkam, Johannesburg, South Africa).

### 3.1. Spectroscopy Measurements

^1^H-, ^11^B- and ^13^C-NMR analysis were performed for all compounds in the study. ^1^H and ^13^C spectra were recorded at 25 °C on a 600 MHz AVANCE II NMR spectrometer (Bruker, Johannesburg, South Africa) at 600.28 MHz and 150.95 MHz respectively. ^11^B-NMR spectra were recorded at 25 °C on a 400 MHz AVANCE III NMR spectrometer (Bruker, Johannesburg, South Africa) at 128.38 MHz. Hydrogen and carbon chemical shifts are relative to hydrogen and carbon in CDCl_3_ at 7.24 ppm and 77.16 ppm, respectively. The following abbreviations are used to describe peak patterns: s = singlet, d = doublet, t = triplet, q = quartet and m = multiplet. UV/vis spectra were recorded on a Cary 5000 UV-Vis-NIR Spectrophotometer (Varian, Johannesburg, South Africa).

### 3.2. Cyclic Voltammetry

All the electrochemical experiments were performed in an Lab Master SP glove box (M Bruan Munich, Germany) under a high purity argon atmosphere (H_2_O and O_2_ < 10 ppm). Cyclic voltammetry (CV) measurements were performed utilising a PARSTAT 2273 potentiostat (Princeton Applied Research, Oak Ridge, TN, USA) running the Powersuite software (Version 2.58). A three-electrode cell was used. A glassy carbon electrode with a surface area 3.14 × 10^−6^ m^2^ was chosen as working electrode, platinum wires were chosen as auxiliary and reference electrodes. The glassy carbon working electrode was polished and prepared before every experiment on a Buhler polishing mat first with 1-micron and then with ¼-micron diamond paste, rinsed with H_2_O, acetone and dichloromethane (DCM), and dried before each experiment. Electrochemical analysis of the complexes was performed in DCM (anhydrous, ≥ 99.8%, contains 40–150 ppm amylene as a stabiliser) at room temperature. Solutions were made in 0.001 dm^3^ spectrochemical grade anhydrous DCM containing ca. 0.0005 M of an analyte, 0.0005 mol dm^−3^ of internal reference (decamethylferrocene, DmFc) and 0.1 mol dm^−3^ of supporting electrolyte tetrabutylammonium tetrakispentafluorophenylborate, [N(*^n^*Bu)_4_][B(C_6_F_5_)_4_] in DCM. Experimental potential data was collected vs. the Pt wire reference electrode but is reported vs. the redox couple of ferrocene, Fc/Fc^+^ at 0 V. *E*°’ (DmFc) = −0.610 V *vs.* Fc/Fc^+^ at 0 V in DCM/[N(*^n^*Bu)_4_][B(C_6_F_5_)_4_]. Scan rates were between 0.05 and 5.00 Vs^−1^. Electrochemical reversibility (or Nernstian behaviour) of redox processes is indicated by a peak current ratio (*i*_pc_/*i*_pa_ for oxidation and *i*_pa_/*i*_pc_ for reduction) of 1 [34,35] and peak current separation Δ*E* = |*E_pa_* − *E_pc_*| = 0.059 V for a one-electron transfer process [36]. In this experiment, due to experimental cell imperfections and ohmic drop effects, Δ*E*_p_ slightly larger than 0.059 V was obtained, even for the known 1 e^-^ transfer processes of decamethylferrocene, DmFc^+^/DmFc, namely 0.074–0.076 V [37,38,39]. The formal reduction potential is determined by *E*°’ = (*E*_pa_ – *E*_pc_)/2 for an electrochemically reversible (and quasi reversible) process where *E*_pa_ (*E*_pc_) = anodic (cathodic) peak potential and *i*_pa_ (*i*_pc_) = anodic (cathodic) peak current.

### 3.3. DFT Calculations

Density functional theory (DFT) optimisations were performed on the neutral and oxidised molecules in the gas phase using the hybrid PBE1PBE [40,41,42] exchange-correlation functional and the triple-ζ basis set 6-311G(d,p) basis set, as implemented in the Gaussian 16 package [43]. Single point calculations using pure BP86 [44,45,46] exchange-correlation were performed in DCM as the solvent, using the IEF-PCM model (integral equation formalism - polarisable continuum model) [47] which solved the non-homogeneous Poisson equation by applying the integral equation formalism (IEF) variant [48]. Time-dependent density-functional theory (TDDFT) calculations were done on the same level of theory. Both the gas phase PBE1PBE/6-311G(d,p) and solvent phase BP86/6-311G(d,p) results gave the same molecular orbital (MO) insight into the observed experimental redox processes. The PBE1PBE exchange-correlation functional previously showed to give good agreement between calculated and experimentally determined bond distances and angles in ferrocene-containing compounds [22,49,50].

### 3.4. Preparationof SubPcs **7**, **8**, **10** and **11**

FcCO_2_H, **1**, FcCH_2_CO_2_H, **2**, Fc(CH_2_)_3_CO_2_H, **4**, FcCO(CH_2_)_2_CO_2_H, **5**, and ClBSubPc(H)_12_, **6**, were synthesised using slight modifications to literature methods (see the Supplementary Materials) [12,13,24]. Compounds **7, 8, 10** and **11** were characterised by NMR, UV/Vis, elemental analysis and melting point (m.p.).

#### 3.4.1. Preparation of FcCO_2_SubPc(H)_12_, **7**

To a solution of chlorosubphthalocyanine, **6**, (200 mg; 0.46 mmol) and dry toluene (3 mLmL), silver trifluoromethanesulfonate (150 mg, 0.58 mmol; 1.25 equivalent (eq.)) was added and the mixture stirred at 45 °C, under argon atmosphere for 4 h. Once the (OTf)SubPc(H)_12_ was generated, ferrocenylcarboxylic acid, **1**, (212 mg, 0.92 mmol, 2 eq.) and *N*,*N*-diisopropylethylamine (0.10 mLmL, 75 mg, 0.58 mmol, 1.25 eq.) was added. The mixture was stirred at 50 °C for 12 h. The solvent was removed by evaporation under reduced pressure and the product was directly purified by flash chromatography using hexane: DCM (1:1) (R_f_: 0.82) as eluent to give 184 mg (63%). m.p.: 172–182 °C, UV/vis: λ_max_ = 563 nm, *ε* = 176547 dm^3^ mol^−1^ cm^−1^ in THF. ^1^H-NMR: δH (600.28 MHz, CDCl3, 25 °C): δ 8.88 (6H, dd, SubPc), 7.90 (6H, dd, SubPc), 3.96 (2H, pt, 2 × CH: Substituted-Cp), 3.95 (2H, pt, 2 × CH: Substituted-Cp), 3.63 (5H, s, Unsubstituted-Cp). ^11^B-NMR: *δ*_B_ (128.38 MHz, CDCl_3_): δ −16.82 (1B). ^13^C- NMR: *δ*_C_ (150.95 MHz, CDCl_3_, 25 °C): δ 172.43 (1C, Fc-CO_2_), 151.67 (6C, SubPc: N-C=N), 131.20 (6C, SubPc: C=C), 130.02 (6C, SubPc: non-peripheral), 122.47 (6C, SubPc: peripheral), 88.08 (1C, Substituted-Cp-ring), 68.51 (5C, Unsubstituted-Cp-ring), 68.04 (2C, Substituted-Cp-ring), 67.11 (2C, Substituted-Cp-ring). Elemental analysis calculated for C_35_H_21_BFeN_6_O_2_ (element, %): C, 67.34; H, 3.39; N, 13.46. Obtained: C, 67.73; H, 3.47; N, 13.58.

#### 3.4.2. Preparation of FcCH_2_CO_2_SubPc(H)_12_, **8**

To a solution of chlorosubphthalocyanine, **6**, (200 mg; 0.46 mmol) and dry toluene (3 mL), silver trifluoromethanesulfonate (150 mg, 0.58 mmol; 1.25 eq.) was added and the mixture stirred at 45 °C, under argon atmosphere for 4 h. Once the (OTf)SubPc(H)_12_ was generated, ferrocenylmethanoic acid, **2**, (225 mg, 0.92 mmol, 2 eq.) and *N*,*N*-diisopropylethylamine (0.10 mL, 75 mg, 0.58 mmol, 1.25 eq.) was added. The mixture was stirred at 50 °C for 12 h. The solvent was removed by evaporation under reduced pressure and the product was directly purified by flash chromatography using hexane: DCM (1:1) (R_f_: 0.78) as eluent to give 140 mg (47%). m.p.: 175–183 °C, UV/vis: λ_max_ = 563 nm, *ε* = 166317 dm^3^ mol^−1^ cm^−1^ in THF. ^1^H-NMR: δH (600.28 MHz, CDCl3, 25 °C): δ 8.86 (6H, dd, SubPc), 7.89 (6H, dd, SubPc), 3.79 (2H, pt, 2 × CH: Substituted-Cp), 3.67 (5H, s, Unsubstituted-Cp), 3.53 (2H, pt, 2 × CH: Substituted-Cp), 2.28 (2H, s, 1 × CH_2_). ^11^B-NMR: *δ*_B_ (128.38 MHz, CDCl_3_): δ −16.76 (1B). ^13^C-NMR: *δ*_C_ (150.95 MHz, CDCl_3_, 25 °C): δ 169.82 (1C, CO), 149.05 (6C, SubPc: N-C=N), 128.59 (6C, SubPc: C=C), 127.41 (6C, SubPc: non-peripheral), 119.85 (6C, SubPc: peripheral), 85.47 (1C, Substituted-Cp-ring), 65.90 (5C, Unsubstituted-Cp-ring), 65.43 (2C, Substituted-Cp-ring), 64.49 (2C, Substituted-Cp-ring), 22.81 (1C, Fc-CH_2_-CO_2_). Elemental analysis calculated for C_36_H_23_BFeN_6_O_2_ (element, %): C, 67.74; H, 3.63; N, 13.17. Obtained: C, 67.90; H, 3.82; N, 13.72.

#### 3.4.3. Preparation of Fc(CH_2_)_3_CO_2_SubPc(H)_12_, **10**

To a solution of chlorosubphthalocyanine, **6**, (200 mg; 0.46 mmol) and dry toluene (3 mL), silver trifluoromethanesulfonate (150 mg, 0.58 mmol; 1.25 eq.) was added and the mixture stirred at 45 °C, under argon atmosphere for 4 h. Once the (OTf)SubPc(H)_12_ was generated, ferrocenylethanoic acid, **4**, (250 mg, 0.92 mmol, 2 eq.) and *N*,*N*-diisopropylethylamine (0.10 mL, 75 mg, 0.58 mmol, 1.25 eq.) was added. The mixture was stirred at 50 °C for 12 h. The solvent was removed by evaporation under reduced pressure and the product was directly purified by flash chromatography using hexane: DCM (1:1) (R_f_: 0.73) as eluent to give 104 mg (43%). m.p.: 182–190 °C, UV/vis: λ_max_ = 563 nm, *ε* = 145673 dm^3^ mol^−1^ cm^−1^ in THF. ^1^H-NMR: δH (600.28 MHz, CDCl3, 25 °C): δ 8.86 (6H, dd, SubPc), 7.88 (6H, dd, SubPc), 3.89 (5H, s, Unsubstituted-Cp), 3.84 (2H, pt, 2 × CH: Substituted-Cp), 3.70 (2H, pt, 2 × CH: Substituted-Cp), 1.76 (2H, s, 1 × CH_2_), 1.26 (2H, s, 1 × CH_2_), 1.06 (2H, s, 1 × CH_2_). ^11^B-NMR: *δ*_B_ (128.38 MHz, CDCl_3_): δ −16.32 (1B). ^13^C-NMR: *δ*_C_ (150.95 MHz, CDCl_3_, 25 °C): δ 172.40 (1C, CO), 151.64 (6C, SubPc: N-C=N), 131.17 (6C, SubPc: C=C), 129.99 (6C, SubPc: non-peripheral), 122.44 (6C, SubPc: peripheral), 88.05 (1C, Substituted-Cp-ring), 68.48 (5C, Unsubstituted-Cp-ring), 68.01 (2C, Substituted-Cp-ring), 67.08 (2C, Substituted-Cp-ring), 28.52 (1C, Fc-CH_2_-CH_2_-CH_2_-CO_2_), 25.40 (1C, Fc-CH_2_-CH_2_-CH_2_-CO_2_), 18.45 (1C, Fc-CH_2_-CH_2_-CH_2_-CO_2_). Elemental analysis calculated for C_38_H_27_BFeN_6_O_2_ (element, %): C, 68.50; H, 4.08; N, 12.61. Obtained: C, 68.61; H, 4.16; N, 12.74.

#### 3.4.4. Preparation of FcCO(CH_2_)_2_CO_2_SubPc(H)_12_, **11**

To a solution of chlorosubphthalocyanine, **6**, (200 mg; 0.46 mmol) and dry toluene (3 mL), silver trifluoromethanesulfonate (150 mg, 0.58 mmol; 1.25 eq.) was added and the mixture stirred at 45 °C, under argon atmosphere for 4 h. Once the (OTf)SubPc(H)_12_ was generated, ferrocenyloxobutanoic acid, **4**, (263 mg, 0.92 mmol, 2 eq.) and *N*,*N*-diisopropylethylamine (0.10 mL, 75 mg, 0.58 mmol, 1.25 eq.) was added. The mixture was stirred at 50 °C for 12 h. The solvent was removed by evaporation under reduced pressure and the product was directly purified by flash chromatography using hexane: DCM (1:1) (R_f_: 0.62) as eluent to give 114 mg (36%). m.p.: 201–207 °C, UV/vis: λ_max_ = 563 nm, *ε* = 113729 dm^3^ mol^−1^ cm^−1^ in THF. ^1^H-NMR: δH (600.28 MHz, CDCl3, 25 °C): δ 8.85 (6H, dd, SubPc), 7.88 (6H, dd, SubPc), 4.49 (2H, pt, 2 × CH: Substituted-Cp), 4.31 (2H, pt, 2 × CH: Substituted-Cp), 3.98 (5H, s, Unsubstituted-Cp), 2.33 (2H, s, 1 × CH_2_), 1.66 (2H, s, 1 × CH_2_). ^11^B-NMR: *δ*_B_ (128.38 MHz, CDCl_3_): δ −16.79 (1B). ^13^C-NMR: *δ*_C_ (150.95 MHz, CDCl_3_, 25 °C): δ 176.47 (1C, Fc-CO), 173.83 (1C, Fc-CO-CH_2_-CH_2_-CO_2_), 155.71 (6C, SubPc: N-C=N), 135.24 (6C, SubPc: C=C), 134.06 (6C, SubPc: non-peripheral), 126.51 (6C, SubPc: peripheral), 92.12 (1C, Substituted-Cp-ring), 72.55 (5C, Unsubstituted-Cp-ring), 72.08 (2C, Substituted-Cp-ring), 71.15 (2C, Substituted-Cp-ring), 32.59 (1C, Fc-CO-CH_2_-CH_2_-CO_2_), 29.47 (1C, Fc-CO-CH_2_-CH_2_-CO_2_). Elemental analysis calculated for C_38_H_25_BFeN_6_O_3_ (element, %): C, 67.09; H, 3.70; N, 12.35. Obtained: C, 67.09; H, 3.82; N, 12.35.

## 4. Conclusions

Subphthalocyanines with ferrocenylcarboxylic acids in the axial position can be synthesized in 63% yields when reactions are performed under strict Schlenk conditions, in this case in a glovebox. The axial ferrocenylcarboxylic moiety did not influence the UV/Vis wavelength maxima of the Soret or Q-bands when comparing ferrocenylcarboxylic containing SubPcs **7**–**11** with parent macrocycle, **6**. The cyclic voltammetry data revealed that the first reversible oxidation process is ferrocene-centered, with the second oxidation and all observed reduction processes are SubPc ring-based. DFT optimization of the oxidized (cation) SubPc was necessary to confirm the locus of the second observed SubPc ring-based oxidation.

The oxidation potential of Fe of the axial ferrocenyl moiety was affected by the electron-rich subphthalocyanine, shifting Fe^II/III^ oxidation potentials with 0.038–0.060 V to a lower oxidation potential compared to Fe^II/III^ oxidation potentials of the free ferrocenylcarboxylic acids. The electron withdrawing ferrocenium moiety in the oxidized axial ligand, withdraws charge from the alkyl chains bonded to it, and consequently the first ring-based oxidation of all ferrocenylsubphthalocyanine dyads Y-BSubPc(H)_12_ **7**–**11** that is influenced by the net electron withdrawing effect of the oxidized axial ferrocenylcarboxylic ligand Y, is exactly the same, namely 0.042 V more positive than *E*°’ of the first ring-based oxidation of Cl-BSubPc(H)_12_ **6** at 0.628 V. The net electron donating effect of the neutral axial ferrocenylcarboxylic ligand Y influenced the ring electron density of the neutral SubPc, leading to a systematic decrease in the two observed ring-based reductions of the ferrocenylsubphthalocyanine dyads as the number of alkyl groups *n* in the different axially bonded ferrocenyl carboxylic acid moieties (Fc-(CH_2_)_n_-CO_2_) increases. Fc(CH_2_)_3_CO_2_BSubPc(H)_12_, **10**, has the lowest first ring-based reduction potential reported to date, due to the strong net electron donating effect of the axial ferrocenylcarboxylic ligand (Fc(CH_2_)_3_COO).

## Figures and Tables

**Table 1:** UV/vis data of SubPcs **6**–**11** in tetrahydrofuran (THF).

	Soret Band	Q Band		
	Max	1st Shoulder	2nd Shoulder	Max
ClBSubPc(H)_12_, **6**, [20]	308	525	532	564
FcCO_2_BSubPc(H)_12_, **7**	299	515	530	563
FcCH_2_CO_2_BSubPc(H)_12_, **8**	300	518	534	563
Fc(CH_2_)_2_CO_2_BSubPc(H)_12_, **9**, [24]	302	505	544	563
Fc(CH_2_)_3_CO_2_BSubPc(H)_12_, **10**	328	521	539	563
FcCO(CH_2_)_2_CO_2_BSubPc(H)_12_, **11**	327	523	542	563

**Table 2:** Cyclic voltammetry data of ligands **1**–**5** and SubPcs **6**–**11** in DCM containing 0.1 mol dm^−3^ [N(*^n^*Bu)_4_][B(C_6_F_5_)_4_] as supporting electrolyte at a scan rate of 0.100 V/s at 25 °C.

	Description	*E_p_* ^a^	*E*°’ (V), Δ*E*_p_ (V)	*i_p_* (μA) ^b^, Current Ratio ^c^
FcCOOH^d^ **1**	Fc	0.321	0.284, 0.074	3.60, 0.99
FcCH_2_COOH ^d^ **2**	Fc	0.047	0.014, 0.066	3.79, 0.99
Fc(CH_2_)_2_COOH ^d^ **3**	Fc	0.020	−0.015, 0.070	3.87, 0.99
Fc(CH_2_)_3_COOH ^d^ **4**	Fc	0.011	−0.024, 0.070	3.98, 0.99
FcCO(CH_2_)_2_COOH ^d^ **5**	Fc	0.330	0.295, 0.070	3.66, 0.99
SubPc **6**	DmFc	−0.647	−0.610, 0.076	3.89, 0.99
ClBSubPc(H)_12_^e^	Wave I	0.674	0.628, 0.086	3.08, 0.99
	Wave II	−1.519	-, -	3.63, -
	Wave III	−2.050	-, -	-, -
SubPc **7**	DmFc	−0.647	−0.610, 0.074	3.91, 0.99
**Fc**CO_2_SubPc(H)_12_	Fc	0.262	0.224, 0.076	3.61, 0.99
(*n* = 0)	Wave I	0.711	0.670, 0.082	3.34, 0.99
	Wave II	−1.643	−1.601, 0.084	3.47, 0.99
	Wave III	−2.124	-, -	-, -
SubPc **8**	DmFc	−0.647	−0.610, 0.075	3.84, 0.99
**Fc**CH_2_CO_2_SubPc(H)_12_	Fc	−0.005	−0.043, 0.076	3.61, 0.99
(*n* = 1)	Wave I	0.710	0.670, 0.080	3.38, 0.99
	Wave II	−1.715	−1.674, 0.082	3.49,0.99
	Wave III	−2.154	-, -	-, -
SubPc **9**	DmFc	−0.647	−0.610,0.074	3.89, 0.99
**Fc**(CH_2_)_2_CO_2_SubPc(H)_12_^f^	Fc	−0.021	−0.058, 0.074	3.66, 0.99
(*n* = 2)	Wave I	0.712	0.670, 0.084	3.31, 0.99
	Wave II	−1.783	−1.741, 0.084	3.42, 0.99
	Wave III	−2.264	-, -	-, -
SubPc **10**	DmFc	−0.647	−0.610, 0.075	3.97, 0.99
**Fc**(CH_2_)_3_CO_2_SubPc(H)_12_	Fc	−0.024	−0.062, 0.076	3.58, 0.99
(*n* = 3)	Wave I	0.711	0.670, 0.082	3.27, 0.99
	Wave II	−1.871	−1.829, 0.084	3.39, 0.99
	Wave III	−2.344	-, -	-, -
SubPc **11**	DmFc	−0.647	−0.610, 0.074	3.84, 0.99
**Fc**CO(CH_2_)_2_CO_2_SubPc(H)_12_	Fc	0.300	0.262, 0.076	3.57, 0.99
	Wave I	0.711	0.670, 0.082	3.35, 0.99
	Wave II	−1.525	−1.482, 0.086	3.41, 0.99
	Wave III	−2.004	-, -	-, -

^a^*E*_p_ is the peak anodic peak for oxidation (*E*_ox_) and peak cathodic peak for reduction (*E*_red_). ^b^ *i*_p_ is the peak anodic peak for oxidation (*i*_pa_) and peak cathodic peak for reduction (*i*_pc_). ^c^ peak current ratio = *i*_pc_/*i*_pa_ for oxidation and *i*_pa_/*i*_pc_ for reduction.^d^ Data from reference [14]. ^e^ Data from reference [20]. ^f^ Data from reference [24].

## Supplementary Materials

The following are available online, Synthesis and characterisation (Section S1, compounds **1**, **2**, **4**– **6**: FcCO_2_H, FcCH_2_CO_2_H, Fc(CH_2_)_3_CO_2_H, FcCO(CH_2_)_2_CO_2_H, ClSubPc(H)_12_), Figures S1–S3: ^1^H, ^13^C and ^11^B NMR of compound **7**; Figure S4–S6: 1H, 13C and 11B NMR of compound **8**; Figures S7–S9: ^1^H, ^13^C and ^11^B NMR of compound **10**; Figures S10–S12: ^1^H, ^13^C and ^11^B NMR of compound **11**; In Section S3 Figures S13–S15: HOMO, LUMO and optimized coordinates of the compounds **7**, **8**, **10** and **11** (respectively).
