# Supplementary material for: Synthesis, Spectroscopy, Electrochemistry and DFT of Electron-Rich Ferrocenylsubphthalocyanines
Source: Molecules. 2020 Jun 1;25(11):2575. doi: 10.3390/molecules25112575 (PMC7321219; doi:10.3390/molecules25112575)
Supplement: Supplementary file 1 [file molecules-25-02575-s001.pdf]

molecules

Supporting Information

Synthesis, Spectroscopy, Electrochemistry and DFT of Electron-rich Ferrocenylsubphthalocyanines

Pieter J. Swarts [1] and Jeanet Conradie [2,*]

1 Department of Chemistry, University of the Free State, Bloemfontein 9300, South Africa
2 Department of Chemistry, UiT – The Arctic University of Norway, Tromsø N-9037, Norway
* Correspondence: conradj@ufs.ac.za; Tel.: +27-(51)-4012194, Fax: +27-4017295

Supporting Information

Contents

Supporting Information 1
1. Synthesis 2
1.1. Preparation of $FcCO_2H$, [1,2]: 2
1.2. Preparation of $FcCH_2CO_2H$, 2 [1,2] 2
1.3. Preparation of Preparation of $Fc(CH_2)_3CO_2H$, 4 [1,2]: 2
1.4. Preparation of $FcCO(CH_2)_2CO_2H$, 5 [2,4]: 3
1.5. Preparation of $ClSubPc(H)_{12}$, 6 [3], Scheme 1: 3
2. NMR 4
2.1. ^{1}H-NMR of $FcCO_2BSubPc(H)_{12}$, **7**: 4
2.2. ^{11}B-NMR of $FcCO_2BSubPc(H)_{12}$, **7**: 4
2.3. ^{13}C-NMR of $FcCO_2BSubPc(H)_{12}$, **7**: 5
2.4. ^{1}H-NMR of Fc $CH_2CO_2BSubPc(H)_{12}$, **8**: 6
2.5. ^{11}B-NMR of Fc $CH_2CO_2BSubPc(H)_{12}$, **8**: 6
2.6. ^{13}C-NMR of Fc $CH_2CO_2BSubPc(H)_{12}$, **8**: 7
2.7. ^{1}H-NMR of $Fc(CH_2)_3CO_2BSubPc(H)_{12}$, **10**: 8
2.8. ^{11}B-NMR of $Fc(CH_2)_3CO_2BSubPc(H)_{12}$, **10**: 8
2.9. ^{13}C-NMR of $Fc(CH_2)_3CO_2BSubPc(H)_{12}$, **10**: 9
2.10. ^{1}H-NMR of $FcCO(CH_2)_2CO_2BSubPc(H)_{12}$, **11**: 10
2.11. ^{11}B-NMR of $FcCO(CH_2)_2CO_2BSubPc(H)_{12}$, **11**: 11
2.12. ^{13}C-NMR of $FcCO(CH_2)_2CO_2BSubPc(H)_{12}$, **11**: 12
3. DFT 13
3.1. LUMO and HOMO of optimized cation species pf SubPc **7**, **10** and **11**. 13
$FcCO_2BSubPc(H)_{12}$, **7**: 13
$Fc(CH_2)_3CO_2BSubPc(H)_{12}$, **10**: 13
$FcCO(CH_2)_2CO_2BSubPc(H)_{12}$, **11**: 14
3.2. Optimized cation coordinates of $FcCO_2BSubPc(H)_{12}$, **7**: 14
3.3. Optimized cation coordinates of $FcCH_2CO_2BSubPc(H)_{12}$, **8**: 15
3.4. Optimized cation coordinates of $Fc(CH_2)_3CO_2BSubPc(H)_{12}$, **10**: 17
3.5. Optimizedcation coordinates of $FcCO(CH_2)_2CO_2BSubPc(H)_{12}$, **11**: 18

4. References 20

1. Synthesis

Free ferrocenylcarboxylic acids **1–5**, were synthesized in multigram quantities using slightly modified methods than previously published [1], as described in our previous publication [2]. The parent macrocycle SubPc **6** was synthesized using previously published methods [3], see Scheme S1.

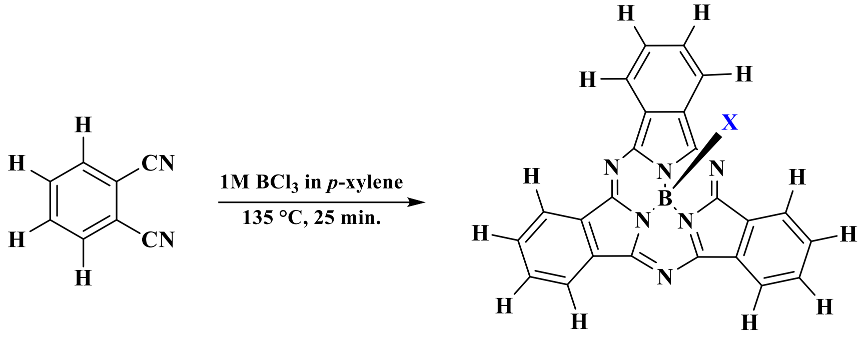

Scheme S1 Reaction scheme of parent macrocycle SubPc, 6.

1.1. Preparation of $FcCO_2H$, [1,2]*:*

2-Chlorobenzoyl ferrocene (1.75 g, 0.005 mol) was added to a mixture of potassium tertiary butoxide (13 g, 0.115 mol) and water (0.61 cm^3, 0.034 mol) in dimethoxyethane (0.1 dm^3) under an argon atmosphere. The mixture produced a yellow slurry which was refluxed for 24 hours. After cooling the mixture, ice water (0.3 dm^3) was added, and the resulting solution was washed with ether (3 × 0.1 dm^3). The aqueous phases were combined and acidified with concentrated hydrochloric acid. The residue was collected by filtration, washed thoroughly with water and air-dried, yielding 1.01 g (80%) of as light-yellow crystals. m.p.: 156–162 °C. ^{1}H-NMR: δH (600.28 MHz, CDCl3, 25 °C): δ 4.84 (2 H, pt, 2 × CH: Substituted-Cp), 4.45 (2 H, pt, 2 × CH: Substituted-Cp), 4.24 (5 H, s, Unsubstituted-Cp). ^{13}C-NMR: δ_C (150.95 MHz, $CDCl_3$, 25 °C): δ 168.24 (1C, C=O), 70.23 (1C, C-CO_2H), 69.72 (5C, Unsubstituted-Cp), 68.42 (2C, Substituted-Cp), 66.24 (2C, Substituted-Cp).

1.2. Preparation of $FcCH_2CO_2H$, 2 [1,2]

To a solution of potassium hydroxide (1 g, 0.018 mol) in water (10 cm^3), a suspension of the ferrocene acetonitrile (0.2 g, 0.00074 mol) in ethanol (5 cm^3) was added and refluxed for 5 h until the evolution of ammonia had ceased. Most (> 95%) of the ethanol was removed under reduced pressure. The residual suspension was dissolved in water (50 cm^3), extracted with ether (2 × 50 cm^3) and filtered. The solution was acidified with 2 M HCl and the precipitate filtered, washed and air-dried to yield 0.110 g (51%) as a white powder. m.p.: 159–165 °C. ^{1}H-NMR: δ_H (600.28 MHz, $CDCl_3$, 25 °C): δ 4.21 (2 H, pt, 2 × CH: Substituted-Cp), 4.13 (5 H, s, Unsubstituted-Cp), 3.73 (2 H, pt, 2 x CH: Substituted-Cp), 3.38 (2H, s, CH_2). ^{13}C-NMR: δ_C (150.95 MHz, $CDCl_3$, 25 °C): δ 172.34 (1C, C=O), 82.44 (1C, C-CO_2H), 69.19 (5C, Unsubstituted-Cp), 68.31 (2C, Substituted-Cp), 67.97 (2C, Substituted-Cp), 39.84 (1C, CH_2).

1.3. Preparation of Preparation of $Fc(CH_2)_3CO_2H$, 4 [1,2]*:*

The ester (0.150 g, 0.00045 mol) was dissolved in ethanol (25 cm^3) followed by the addition of sodium hydroxide solution (25 cm^3, 2 M). The solution was stirred for 1 hour at room temperature followed by the addition of ice (25 m^3) and washed with cold diethyl ether (3 × 50 cm^3). While cooling the solution by adding fresh ice chunks, the water phase was acidified with 1 M HCl and the precipitate filtered, washed and air-dried to liberate 0.132 g (93%) as an off-white powder. m.p.: 120–124 °C. ^{1}H-NMR: δ_H (600.28 MHz, $CDCl_3$, 25 °C): δ 4.12 (5 H, s, Unsubstituted-Cp), 4.09 (2 H, pt,

2 x CH: Substituted-Cp), 4.07 (2 H, pt, 2 x CH: Substituted-Cp), 2.38 (2H, d, CH_2), 1.84 (2H, d, CH_2), 0.86 (2H, m, CH_2). ^{13}C-NMR: δ_C (150.95 MHz, $CDCl_3$, 25 °C): δ 179.42 (1C, C=O), 82.44 (1C, C-CO_2H), 68.31 (2C, Substituted-Cp), 67.48 (5C, Unsubstituted-Cp), 66.24 (2C, Substituted-Cp), 33.45 (1C, CH_2-CH_2-CH_2), 28.88 (1C, CH_2-CH_2-CH_2), 33.45 (1C, CH_2-CH_2-CH_2).

1.4. Preparation of FcCO(CH_2)$_2$$CO_2$H, 5 [2,4]*:*

Succinic anhydride (0.250 g, 0.00215 mol) dissolved in dichloromethane (25 cm^3) was added to a mixture of ferrocene (0.250 g, 0.0215 mol) and aluminium chloride (0.76 g, 0.0056 mol) in dichloromethane (25 cm^3) under a nitrogen atmosphere. The reaction mixture was refluxed for 24 hours. After cooling, ice-cold water (40 cm^3) was added and the aqueous layer extracted twice with dichloromethane. The combined dichloromethane extracts were thoroughly washed with water. The organic phase was then extracted twice with equal amounts of 2 M NaOH. While cooling the solution with ice, the water phase was acidified with 1 M HCl and the precipitate filtered, washed with water and air-dried to liberate 1.1 g (74%) as orange crystals. m.p.: 134–148 °C. ^{1}H-NMR: δ_H (600.28 MHz, $CDCl_3$, 25 °C): δ 4.80 (2 H, pt, 2 × CH: Substituted-Cp), 4.51 (2 H, pt, 2 × CH: Substituted-Cp), 4.22 (5 H, s, Unsubstituted-Cp), 3.07 (2H, d, CH_2), 2.75 (2H, d, CH_2). ^{13}C-NMR: δ_C (150.95 MHz, $CDCl_3$, 25 °C): δ 202.54 (1C, C=O), 171.21 (1C, CO_2H), 80.38 (1C, C-CO_2H), 72.55 (2C, Substituted-Cp), 70.14 (5C, Unsubstituted-Cp), 69.41 (2C, Substituted-Cp), 33.45 (2C, CH_2-CH_2), 28.88 (2C, CH_2-CH_2) .

1.5. Preparation of ClSubPc(H)$_{12}$, 6 [3], Scheme S1:

BCl_3 (15 cm^3,1 M solution in p-xylene, 1.5 eq.) was added to dry phthalonitrile (1 g, 0.008 mol) in a glove box (H_2O: < 0.5 ppm and O_2: < 10 ppm) at room temperature in a high-pressure glass tube. The reaction mixture was stirred under reflux (137 °C) for 30 minutes. The solvent was evaporated and the solid was extracted with toluene (0.4 dm^3). The solution was evaporated, and the resultant purple solid was thoroughly washed with methanol (0.2 dm^3) and hexane (0.2 dm^3). Pure ClSubPc(H)$_{12}$ was obtained as a purple solid, yield: 94% (0.94 g). MP: 375–380 °C. ^{1}H-NMR: δ_H (600.28 MHz, $CDCl_3$): δ 8.88 (6H, q, non-peripheral H_6) and 7.94 (6H, q, peripheral H_6). ^{11}B-NMR: δ_B (128.38 MHz, $CDCl_3$): δ −16.22 (1B). ^{13}C-NMR: δ_C (150.95 MHz, $CDCl_3$, 25 °C): δ 149.68 (6C, C=N: inner core carbons), 125.68 (6C, C=C: iminoisoindoline unit), 122.01 (6C, non-peripheral C_6), 119.84 (6C, peripheral C_6). IR: υ/cm^{-1}: 1451 (C=C, Stretch). Elemental analysis calculated C, 66.94; H, 2.81; N, 19.51, obtained: C, 66.42; H, 2.68; N, 18.31.

2. NMR

2.1. ^{1}H-NMR of FcCO2BSubPc(H)12, **7**:

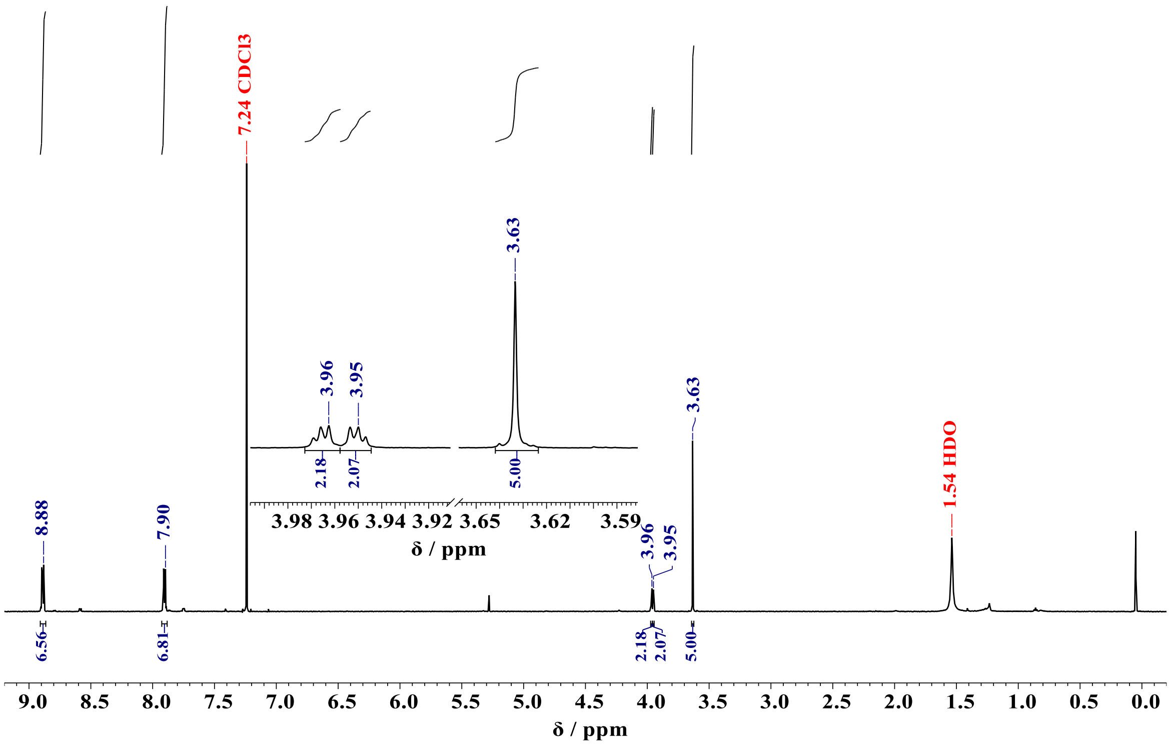

Figure S1. ^{1}H-NMR of $FcCO_2BSubPc(H)_{12}$, **7.**

^{1}H-NMR: δH (600.28 MHz, CDCl3, 25 °C): δ 8.88 (6H, dd, SubPc), 7.90 (6H, dd, SubPc), 3.96 (2H, pt, 2 x CH: Substituted-Cp), 3.95 (2H, pt, 2 x CH: Substituted-Cp), 3.63 (5H, s, Unsubstituted-Cp).

2.2. ^{11}B-NMR of FcCO2BSubPc(H)12, **7**:

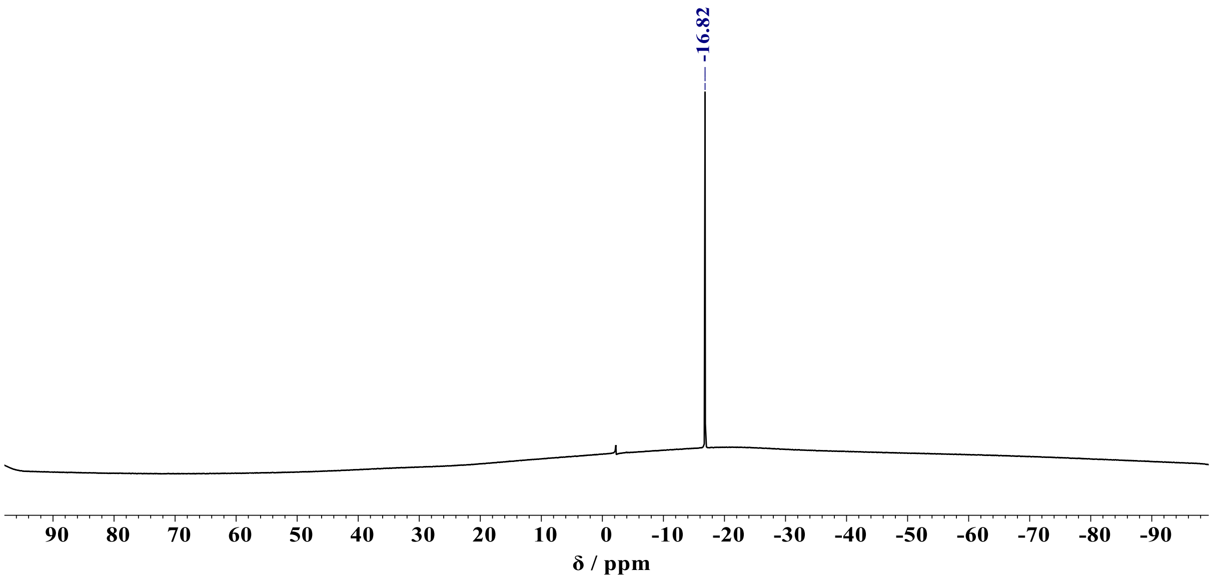

Figure S2. ^{11}B-NMR of $FcCO_2BSubPc(H)_{12}$, **7.**

^{11}B-NMR: δ_B (128.38 MHz, $CDCl_3$): δ -16.82 (1B).

2.3. ¹³C-NMR of $FcCO_2BSubPc(H)_{12}$, **7**:

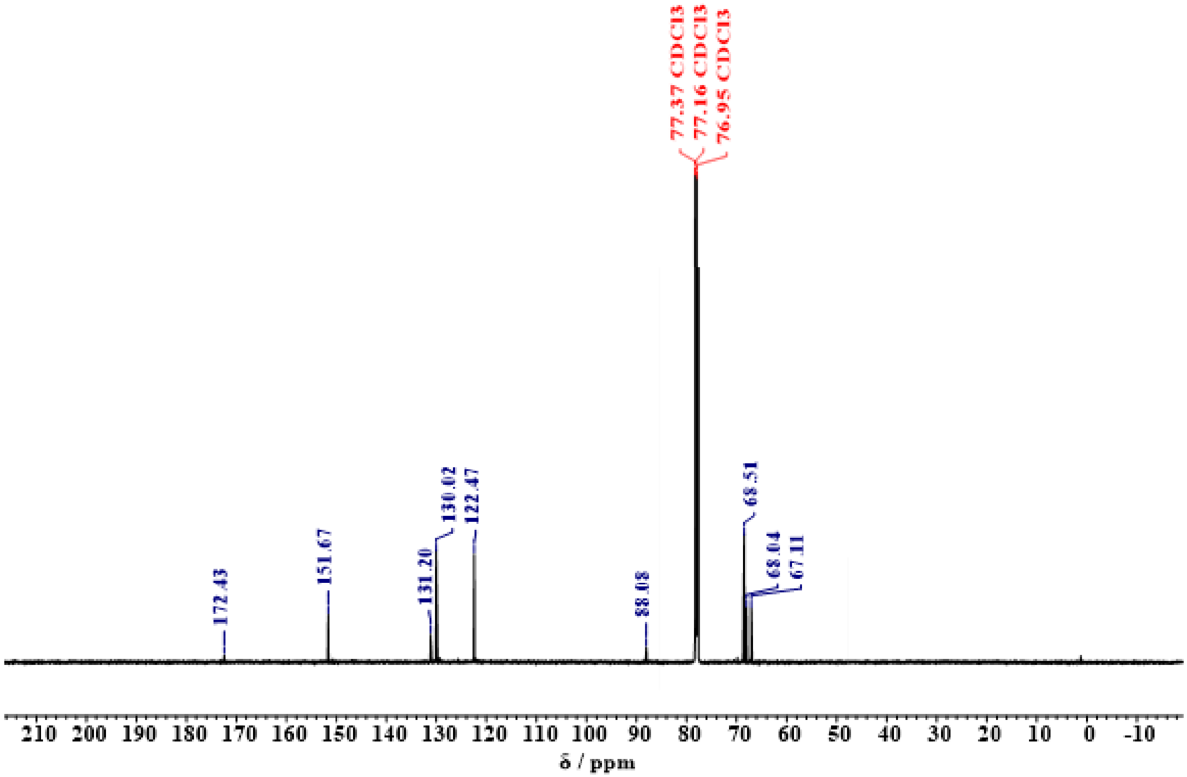

Figure S3. ¹³C-NMR of $FcCO_2BSubPc(H)_{12}$, **7.**

¹³C-NMR: δ_C (150.95 MHz, $CDCl_3$, 25 °C): δ 172.43 (1C, Fc-$\underline{C}O_2$), 151.67 (6C, SubPc: N-$\underline{C}$=N), 131.20 (6C, SubPc: C=C), 130.02 (6C, SubPc: non-peripheral), 122.47 (6C, SubPc: peripheral), 88.08 (1C, Substituted-Cp-ring), 68.51 (5C, Unsubstituted-Cp-ring), 68.04 (2C, Substituted-Cp-ring), 67.11 (2C, Substituted-Cp-ring).

2.4. ^{1}H-NMR of Fc $CH_2CO_2BSubPc(H)_{12}$, **8**:

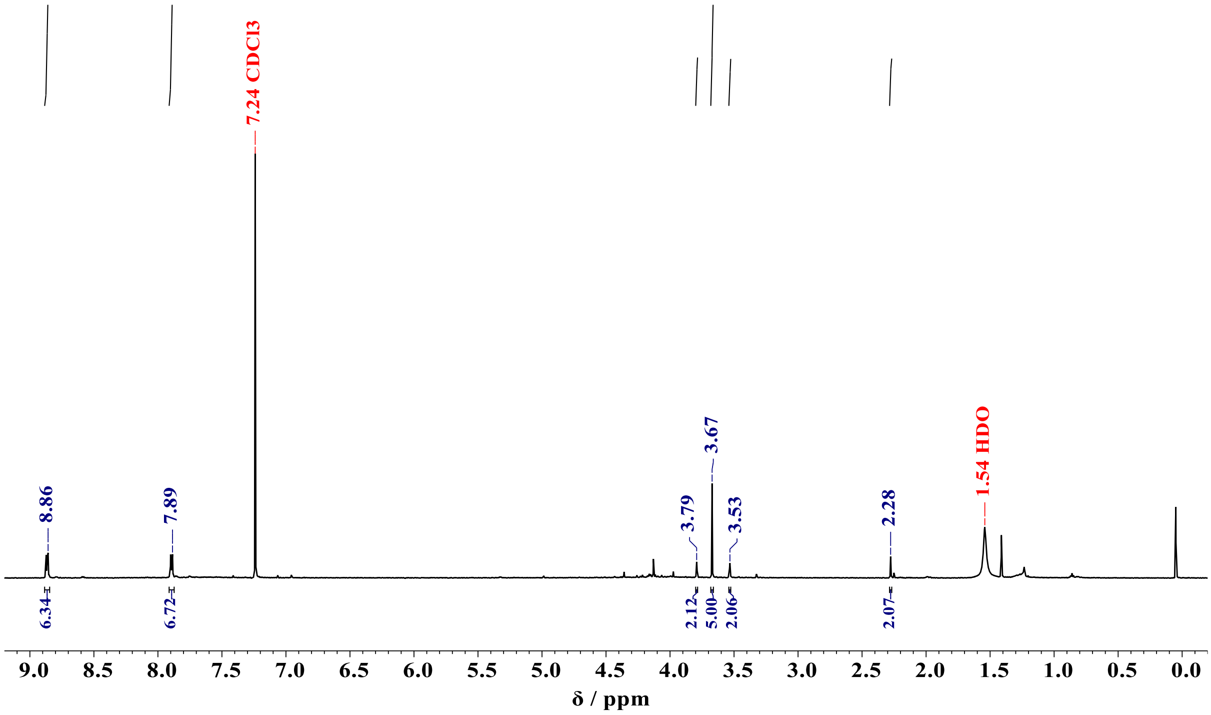

Figure S4. 1H-NMR of Fc CH2CO2BSubPc(H)12, 8.

^{1}H-NMR: δH (600.28 MHz, CDCl3, 25 °C): δ 8.86 (6H, dd, SubPc), 7.89 (6H, dd, SubPc), 3.79 (2H, pt, 2 x CH: Substituted-Cp), 3.67 (5H, s, Unsubstituted-Cp), 3.53 (2H, pt, 2 x CH: Substituted-Cp), 2.28 (2H, s, 1 x CH_2).

2.5. ^{11}B-NMR of Fc $CH_2CO_2BSubPc(H)_{12}$, **8**:

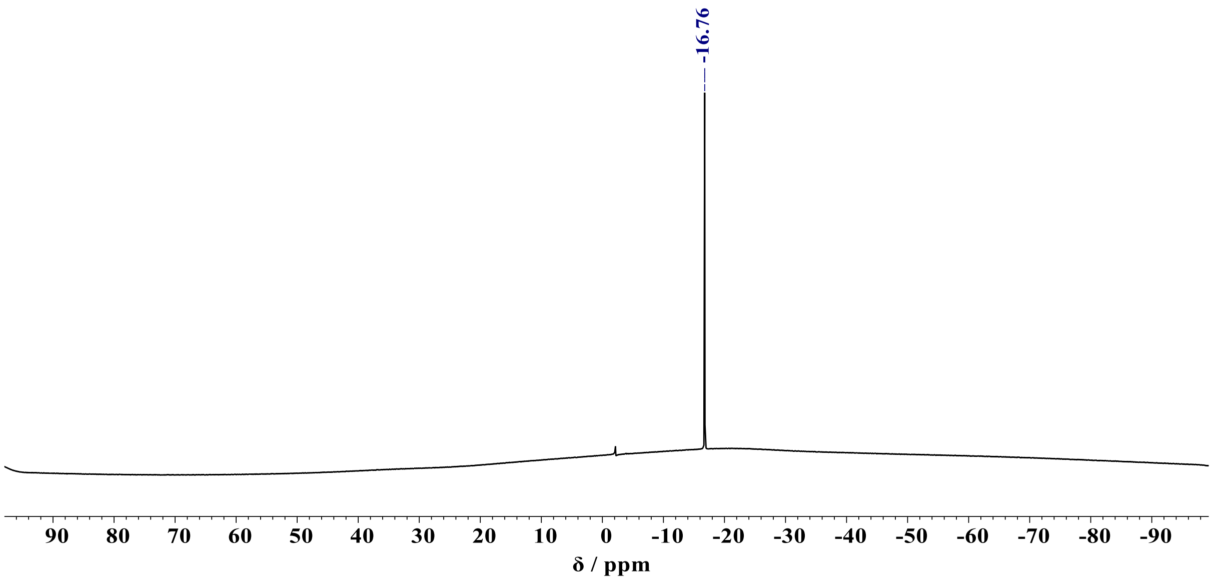

Figure S5. ^{11}B-NMR of Fc CH2CO2BSubPc(H)12, **8**.

^{11}B-NMR: δ_B (128.38 MHz, $CDCl_3$): δ -16.76 (1B).

2.6. ^{13}C-NMR of Fc $CH_2CO_2BSubPc(H)_{12}$, **8**:

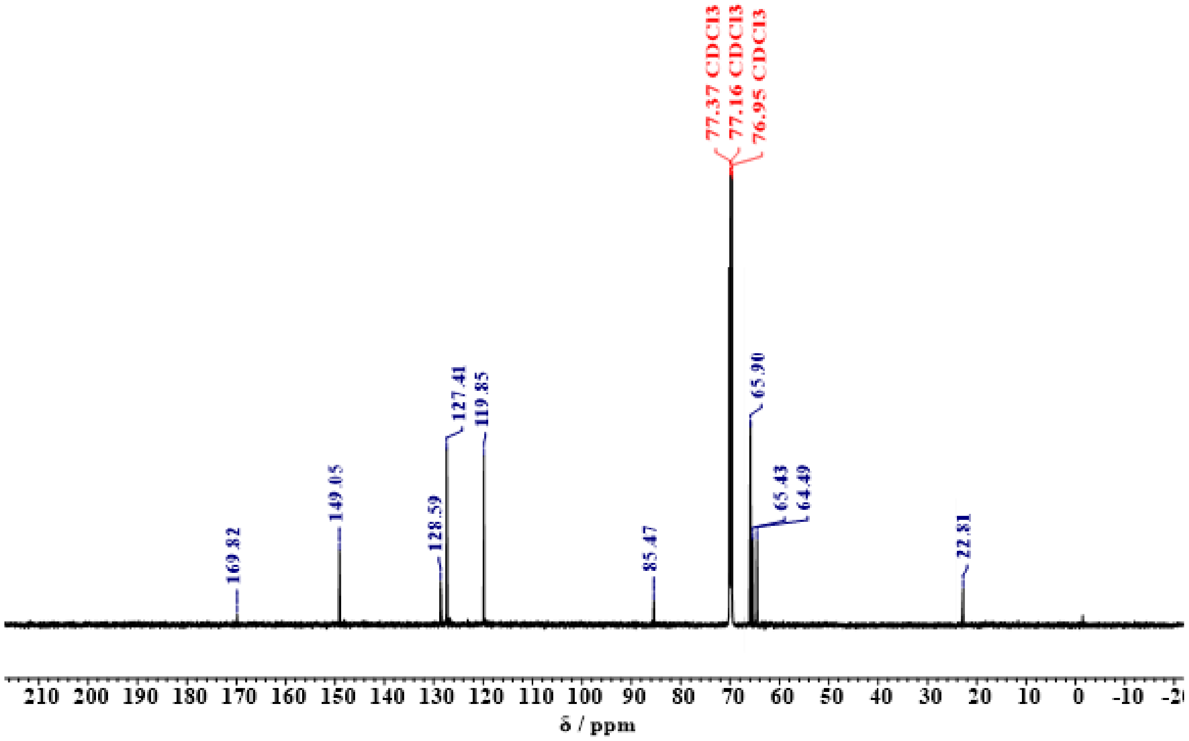

Figure S6. ^{13}C-NMR of Fc CH2CO2BSubPc(H)12, **8**.

^{13}C-NMR: δ_C (150.95 MHz, $CDCl_3$, 25 °C): δ 169.82 (1C, C̲O), 149.05 (6C, SubPc: N-C̲=N), 128.59 (6C, SubPc: C=C), 127.41 (6C, SubPc: non-peripheral), 119.85 (6C, SubPc: peripheral), 85.47 (1C, Substituted-Cp-ring), 65.90 (5C, Unsubstituted-Cp-ring), 65.43 (2C, Substituted-Cp-ring), 64.49 (2C, Substituted-Cp-ring), 22.81 (1C, Fc-C̲H_2-CO_2).

2.7. ^{1}H-NMR of $Fc(CH_2)_3CO_2BSubPc(H)_{12}$, **10**:

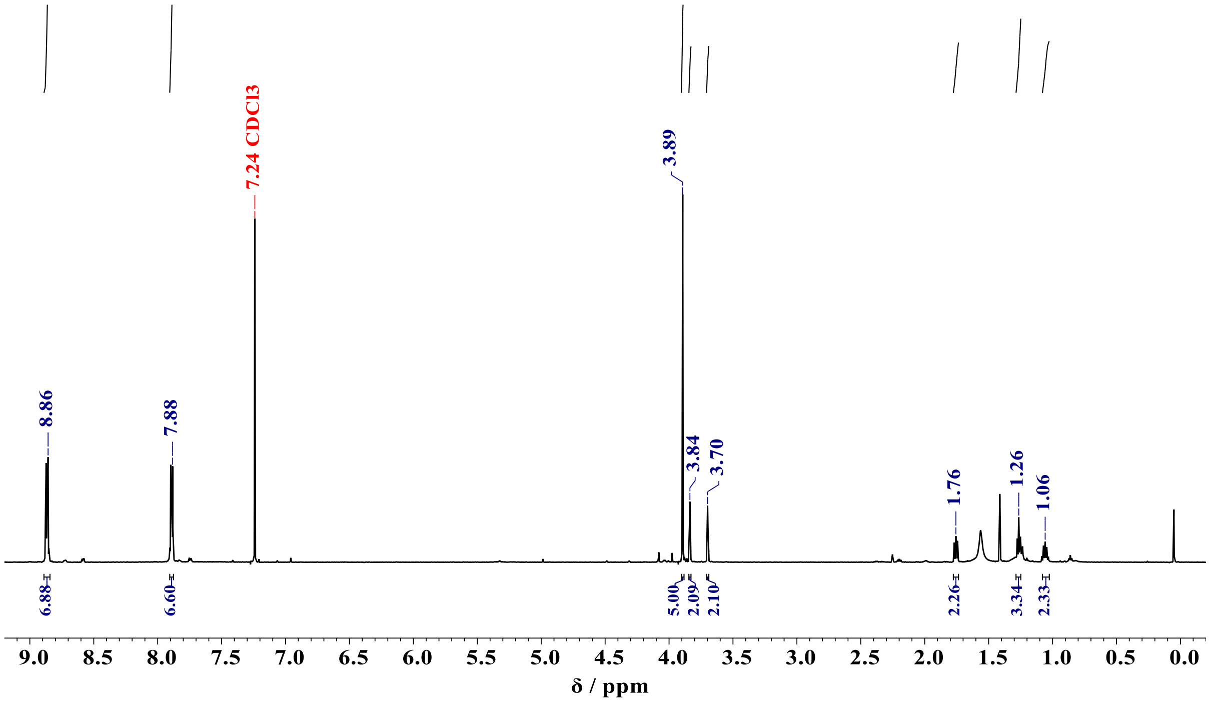

Figure S7. ^{1}H-NMR of Fc(CH2)3CO2BSubPc(H)12, **10**.

^{1}H-NMR: δH (600.28 MHz, CDCl3, 25 °C): δ 8.86 (6H, dd, SubPc), 7.88 (6H, dd, SubPc), 3.89 (5H, s, Unsubstituted-Cp), 3.84 (2H, pt, 2 x CH: Substituted-Cp), 3.70 (2H, pt, 2 x CH: Substituted-Cp), 1.76 (2H, s, 1 x CH_2), 1.26 (2H, s, 1 x CH_2), 1.06 (2H, s, 1 x CH_2).

2.8. ^{11}B-NMR of $Fc(CH_2)_3CO_2BSubPc(H)_{12}$, **10**:

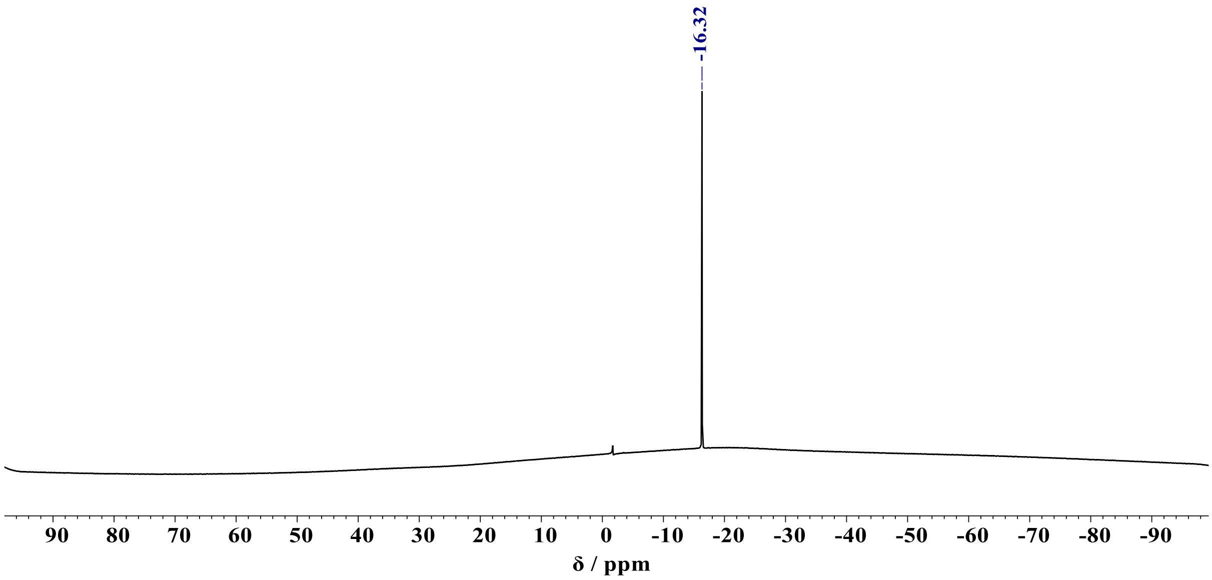

Figure S8. ^{11}B-NMR of Fc(CH2)3CO2BSubPc(H)12, **10**.

^{11}B-NMR: δ_B (128.38 MHz, $CDCl_3$): δ -16.32 (1B).

2.9. ^{13}C-NMR of Fc(CH$_2$)$_3$CO$_2$BSubPc(H)$_{12}$, **10**:

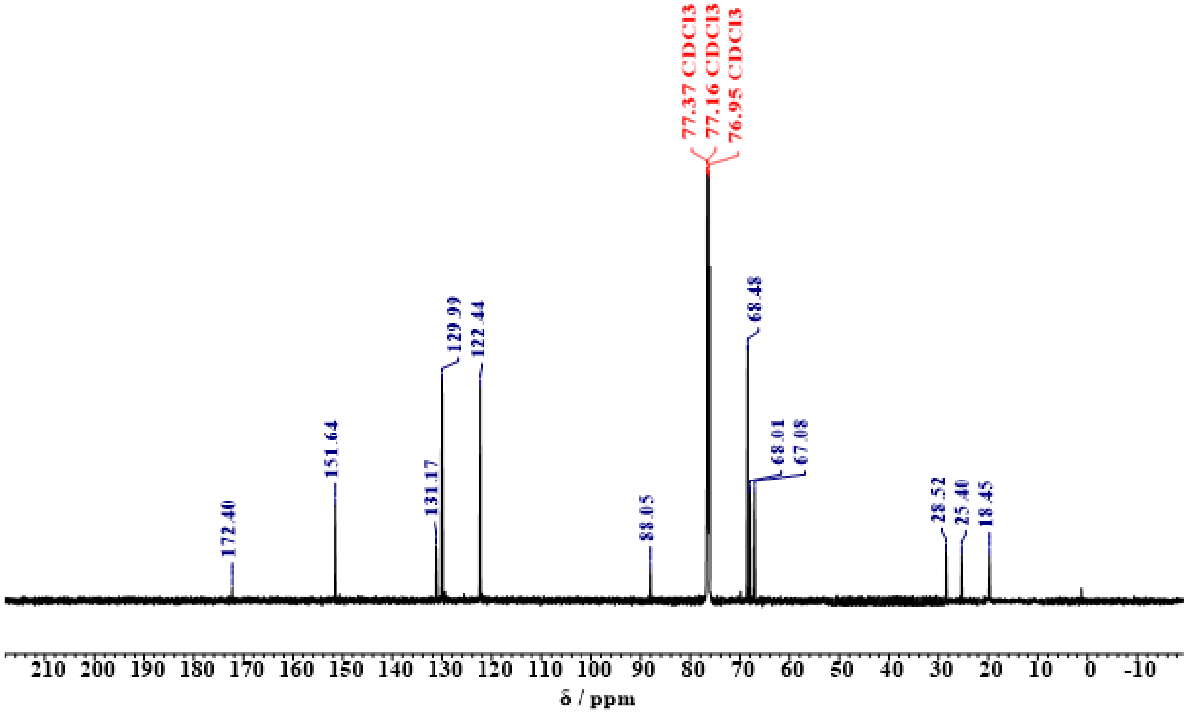

Figure 9. ^{13}C-NMR of Fc(CH2)3CO2BSubPc(H)12, **10**.

^{13}C-NMR: δ_C (150.95 MHz, CDCl$_3$, 25 °C): δ 172.40 (1C, C̲O), 151.64 (6C, SubPc: N-C̲=N), 131.17 (6C, SubPc: C=C), 129.99 (6C, SubPc: non-peripheral), 122.44 (6C, SubPc: peripheral), 88.05 (1C, Substituted-Cp-ring), 68.48 (5C, Unsubstituted-Cp-ring), 68.01 (2C, Substituted-Cp-ring), 67.08 (2C, Substituted-Cp-ring), 28.52 (1C, Fc-CH$_2$-CH$_2$-$\underline{CH_2}$-CO$_2$), 25.40 (1C, Fc-CH$_2$-$\underline{CH_2}$-CH$_2$-CO$_2$), 18.45 (1C, Fc-$\underline{CH_2}$-CH$_2$-CH$_2$-CO$_2$).

2.10. *^{1}H-NMR of FcCO(CH$_2$)$_2$CO$_2$BSubPc(H)$_{12}$,* **11**:

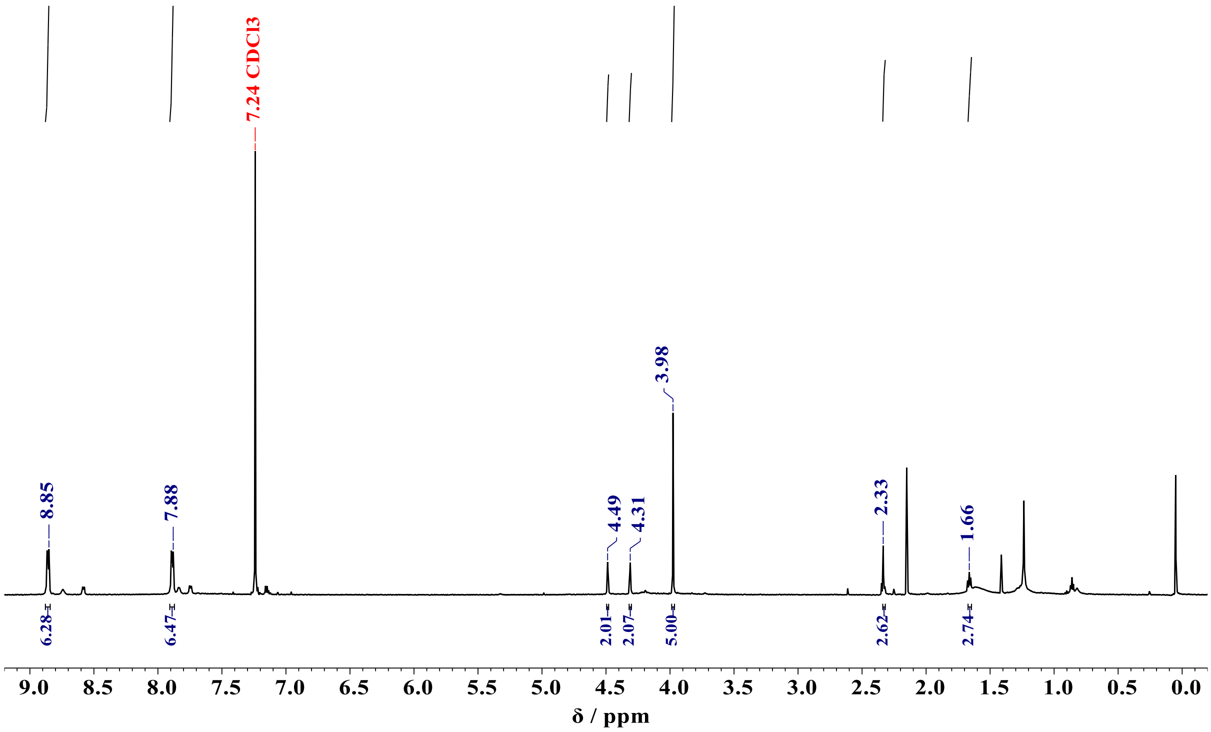

Figure S10. ^{1}H-NMR of FcCO(CH2)2CO2BSubPc(H)12, **11**.

^{1}H-NMR: δH (600.28 MHz, CDCl3, 25 °C): δ 8.85 (6H, dd, SubPc), 7.88 (6H, dd, SubPc), 4.49 (2H, pt, 2 x CH: Substituted-Cp), 4.31 (2H, pt, 2 x CH: Substituted-Cp), 3.98 (5H, s, Unsubstituted-Cp), 2.33 (2H, s, 1 x CH$_2$), 1.66 (2H, s, 1 x CH$_2$).

2.11. ^{11}*B-NMR of FcCO(CH*$_2$*)*$_2$*CO*$_2$*BSubPc(H)*$_{12}$*,* **11**:

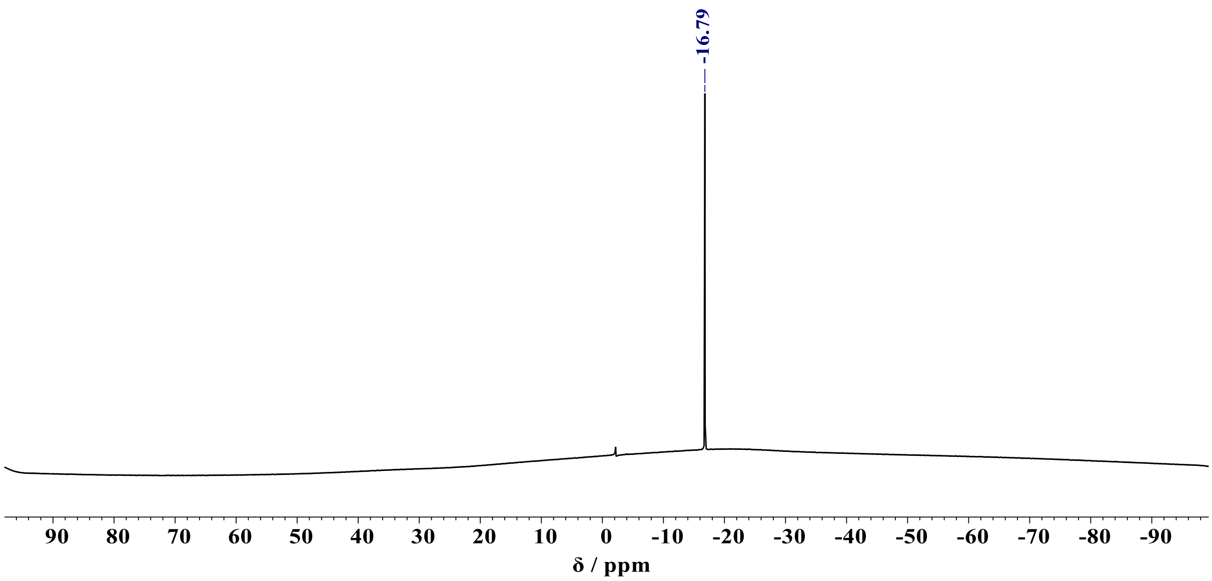

Figure S11. ^{11}B-NMR of FcCO(CH2)2CO2BSubPc(H)12, **11**.

^{11}B-NMR: δ_B (128.38 MHz, $CDCl_3$): δ -16.79 (1B).

2.12. ^{13}C-NMR of FcCO(CH$_2$)$_2$CO$_2$BSubPc(H)$_{12}$, **11**:

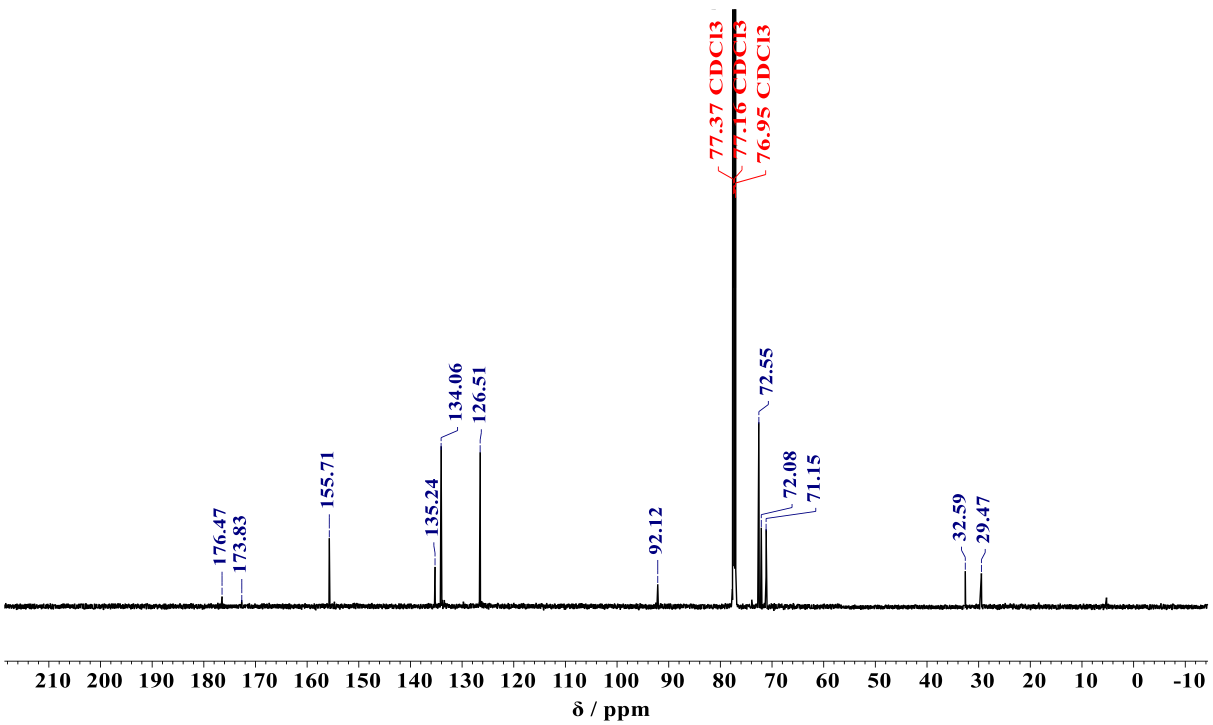

Figure S12. ^{13}C-NMR of FcCO(CH2)2CO2BSubPc(H)12, **11.**

^{13}C-NMR: δ_C (150.95 MHz, $CDCl_3$, 25 °C): δ 176.47 (1C, Fc-$\underline{C}$O), 173.83 (1C, Fc-CO-CH_2-CH_2-$\underline{C}O_2$), 155.71 (6C, SubPc: N-$\underline{C}$=N), 135.24 (6C, SubPc: C=C), 134.06 (6C, SubPc: non-peripheral), 126.51 (6C, SubPc: peripheral), 92.12 (1C, Substituted-Cp-ring), 72.55 (5C, Unsubstituted-Cp-ring), 72.08 (2C, Substituted-Cp-ring), 71.15 (2C, Substituted-Cp-ring), 32.59 (1C, Fc-CO-CH_2-$\underline{CH_2}$-CO_2), 29.47 (1C, Fc-CO-$\underline{CH_2}$-CH_2-CO_2).

3. DFT

3.1. LUMO and HOMO of optimized cation species pf SubPc **7**, **10** *and* **11**.

$FcCO_2BSubPc(H)_{12}$, **7**:

LUMO	HOMO
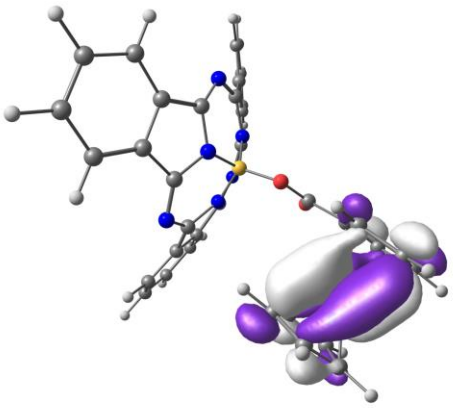	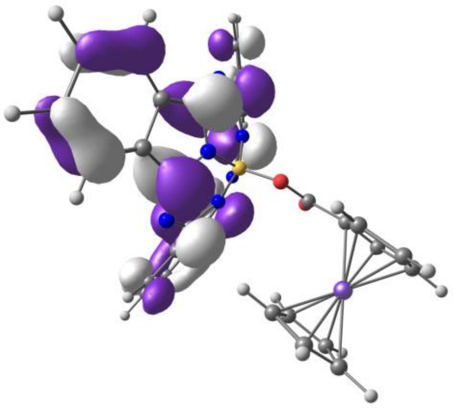

Figure 13. LUMO and HOMO of optimized cation species of SubPc **7.**

A contour of 0.03 e/Å^3 was used for the orbital plots. Colour code of atoms (online version): Fe (purple), B (yellow), C (grey), O (red), H (white).

$Fc(CH_2)_3CO_2BSubPc(H)_{12}$, **10**:

LUMO	HOMO
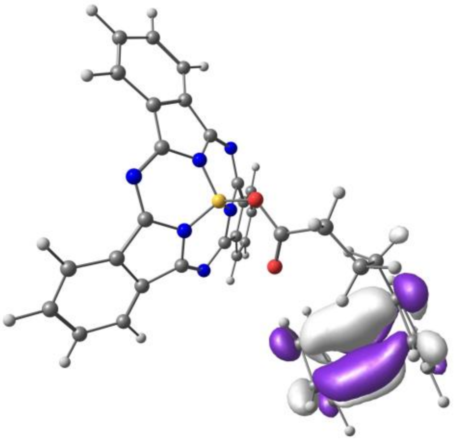	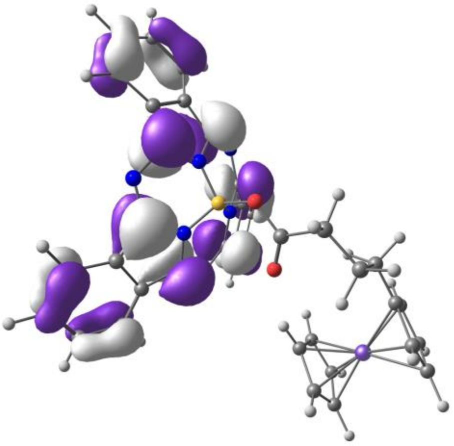

Figure S14. LUMO and HOMO of optimized cation species of SubPc **10.**

A contour of 0.03 e/Å^3 was used for the orbital plots. Colour code of atoms (online version): Fe (purple), B (yellow), C (grey), O (red), H (white).

FcCO(CH$_2$)$_2$CO$_2$BSubPc(H)$_{12}$, **11**:

LUMO	HOMO
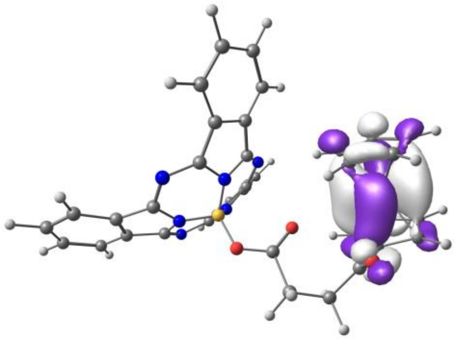	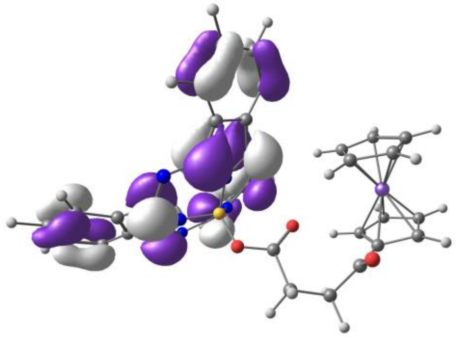

Figure S15. LUMO and HOMO of optimized cation species of SubPc **11.**

A contour of 0.03 e/Å^3 was used for the orbital plots. Colour code of atoms (online version): Fe (purple), B (yellow), C (grey), O (red), H (white).

3.2. Optimized cation coordinates of FcCO$_2$BSubPc(H)$_{12}$, **7**:

Fe	4.097544000	-0.060142000	-1.082864000
O	0.421510000	0.028743000	-1.294316000
O	0.991721000	-2.137841000	-1.032484000
N	-1.906289000	-0.843176000	-0.789395000
N	-1.547142000	-2.494904000	0.868678000
N	-0.435734000	-0.412149000	1.008685000
N	-0.229187000	1.812239000	1.797597000
N	-1.291875000	1.374964000	-0.266953000
N	-3.158202000	0.972357000	-1.655135000
C	-2.972211000	-0.333232000	-1.476795000
C	-3.906055000	-1.434646000	-1.615439000
C	-3.406454000	-2.509837000	-0.831017000
C	-2.170342000	-2.058541000	-0.220250000
C	-5.139807000	-1.531481000	-2.250446000
H	-5.528445000	-0.701967000	-2.829797000
C	-5.846410000	-2.716495000	-2.120216000
H	-6.803146000	-2.825898000	-2.618925000
C	-5.353647000	-3.777146000	-1.346907000
H	-5.939001000	-4.686163000	-1.262693000
C	-4.141046000	-3.681549000	-0.682754000
H	-3.767772000	-4.491679000	-0.067061000
C	-0.747956000	-1.641889000	1.508195000
C	-0.311870000	-1.614691000	2.888905000
C	0.104499000	-0.282110000	3.176814000
C	-0.085960000	0.495109000	1.968850000
C	-0.342158000	-2.580639000	3.892170000
H	-0.682625000	-3.586612000	3.674993000
C	0.068875000	-2.215329000	5.162740000
H	0.065513000	-2.951854000	5.958734000

C	0.475279000	-0.902228000	5.447615000
H	0.776290000	-0.650187000	6.458623000
C	0.482783000	0.077206000	4.469023000
H	0.767820000	1.098303000	4.696080000
C	-0.909738000	2.226976000	0.726718000
C	-1.653843000	3.448334000	0.488671000
C	-2.563536000	3.185591000	-0.575915000
C	-2.365324000	1.807950000	-0.980826000
C	-1.677168000	4.671368000	1.153067000
H	-1.003298000	4.862391000	1.980445000
C	-2.583813000	5.628274000	0.727247000
H	-2.612192000	6.594230000	1.219472000
C	-3.477610000	5.369892000	-0.322431000
H	-4.178697000	6.141150000	-0.621793000
C	-3.488584000	4.146889000	-0.972630000
H	-4.193708000	3.936166000	-1.768463000
C	1.194044000	-1.020582000	-1.437584000
C	2.466754000	-0.678415000	-2.142791000
C	2.882859000	0.596036000	-2.631561000
H	2.293272000	1.500864000	-2.592508000
C	4.198124000	0.456236000	-3.136894000
H	4.810639000	1.250302000	-3.542539000
C	4.600083000	-0.891804000	-2.965314000
H	5.570040000	-1.298147000	-3.218479000
C	3.536718000	-1.597666000	-2.352912000
H	3.519403000	-2.640028000	-2.066518000
C	3.592728000	0.864357000	0.757792000
H	2.652638000	1.365028000	0.952699000
C	4.753199000	1.453689000	0.198509000
H	4.859014000	2.490299000	-0.090149000
C	5.737837000	0.437464000	0.049786000
H	6.733757000	0.569210000	-0.349171000
C	5.173171000	-0.784552000	0.517591000
H	5.659571000	-1.749837000	0.529693000
C	3.847886000	-0.514107000	0.952233000
H	3.133953000	-1.241888000	1.315430000
B	-0.764771000	-0.000623000	-0.374602000

3.3. Optimized cation coordinates of $FcCH_2CO_2BSubPc(H)_{12}$, **8**:

Fe	-5.638214000	-0.003938000	-0.444545000
N	2.287587000	0.491683000	-1.283334000
N	2.653924000	2.625992000	-0.339463000
N	1.538397000	0.948198000	0.907439000
N	1.553165000	-0.834221000	2.465130000
N	1.677382000	-1.284707000	0.144958000
N	2.924862000	-1.718393000	-1.821753000
C	2.948125000	-0.407342000	-2.064551000
C	3.837669000	0.383826000	-2.891718000
C	3.753575000	1.729023000	-2.433051000
C	2.813480000	1.747505000	-1.329733000
C	4.739463000	0.030809000	-3.891433000

H	4.817447000	-0.998070000	-4.223638000
C	5.525418000	1.029195000	-4.442907000
H	6.225480000	0.783200000	-5.233866000
C	5.442653000	2.354562000	-3.990933000
H	6.080399000	3.107359000	-4.441271000
C	4.571786000	2.715407000	-2.976023000
H	4.522045000	3.734494000	-2.609928000
C	2.101457000	2.188063000	0.791068000
C	2.247989000	2.673457000	2.148627000
C	1.910548000	1.598792000	3.017824000
C	1.557213000	0.466748000	2.185053000
C	2.729943000	3.875227000	2.660352000
H	3.010506000	4.683405000	1.994605000
C	2.839095000	4.001156000	4.035189000
H	3.198670000	4.931946000	4.460216000
C	2.506269000	2.941406000	4.892576000
H	2.615490000	3.073835000	5.963489000
C	2.055876000	1.729488000	4.396254000
H	1.822264000	0.900355000	5.054311000
C	1.690152000	-1.683964000	1.450748000
C	2.196675000	-3.041874000	1.433406000
C	2.619010000	-3.317755000	0.103387000
C	2.369912000	-2.124578000	-0.680934000
C	2.404598000	-3.969559000	2.450090000
H	2.103729000	-3.746697000	3.467348000
C	3.003328000	-5.174360000	2.120885000
H	3.163909000	-5.920334000	2.891621000
C	3.419881000	-5.446226000	0.809060000
H	3.894012000	-6.397053000	0.591328000
C	3.248282000	-4.520102000	-0.206438000
H	3.591111000	-4.717567000	-1.215650000
C	-3.579526000	-0.580734000	-0.565948000
C	-4.057941000	-0.682775000	0.766938000
H	-3.639630000	-0.158849000	1.613684000
C	-5.161127000	-1.576655000	0.785517000
H	-5.734845000	-1.856304000	1.658060000
C	-5.382287000	-2.027120000	-0.546354000
H	-6.149747000	-2.715150000	-0.871958000
C	-4.410855000	-1.400816000	-1.376363000
H	-4.318448000	-1.518048000	-2.447869000
C	-6.120336000	1.606635000	-1.652715000
H	-5.543498000	1.916358000	-2.512963000
C	-5.919074000	2.028460000	-0.307644000
H	-5.168388000	2.724567000	0.039325000
C	-6.887317000	1.371752000	0.501978000
H	-6.986821000	1.461421000	1.574740000
C	-7.683199000	0.556518000	-0.340545000
H	-8.479937000	-0.100155000	-0.018878000
C	-7.210965000	0.697982000	-1.666050000
H	-7.590917000	0.174110000	-2.532347000
B	1.290929000	0.083652000	-0.269727000
C	-1.155266000	-0.074507000	-0.201265000

O	-0.074413000	0.208442000	-0.879421000
O	-1.209245000	-0.474132000	0.935661000
C	-2.411165000	0.219966000	-1.027342000
H	-2.202190000	0.045902000	-2.083569000
H	-2.589718000	1.295388000	-0.912792000

3.4. Optimized cation coordinates of $Fc(CH_2)_3CO_2BSubPc(H)_{12}$, **10**:

Fe	5.213439000	0.013784000	-0.271772000
N	-3.096825000	-0.591163000	-0.878862000
N	-2.803519000	-2.605705000	0.317931000
N	-1.250533000	-0.834113000	0.565828000
N	-0.454006000	1.101260000	1.673628000
N	-1.864218000	1.307150000	-0.215761000
N	-4.006258000	1.564797000	-1.193096000
C	-4.111296000	0.237132000	-1.247326000
C	-5.281708000	-0.612963000	-1.360802000
C	-4.909263000	-1.904550000	-0.894308000
C	-3.514846000	-1.831198000	-0.499314000
C	-6.596099000	-0.342225000	-1.726793000
H	-6.881332000	0.648078000	-2.062742000
C	-7.518735000	-1.373510000	-1.651504000
H	-8.546651000	-1.193963000	-1.946887000
C	-7.151966000	-2.646132000	-1.191640000
H	-7.903421000	-3.426410000	-1.140174000
C	-5.852961000	-2.921610000	-0.794945000
H	-5.572084000	-3.899106000	-0.420049000
C	-1.733807000	-2.064942000	0.903235000
C	-1.083392000	-2.403960000	2.152599000
C	-0.360166000	-1.252494000	2.575402000
C	-0.571373000	-0.223162000	1.578231000
C	-1.154275000	-3.534093000	2.963629000
H	-1.727964000	-4.399072000	2.650753000
C	-0.478767000	-3.515129000	4.172159000
H	-0.509549000	-4.387223000	4.816183000
C	0.230755000	-2.378954000	4.591970000
H	0.730077000	-2.394052000	5.554847000
C	0.283715000	-1.236297000	3.811266000
H	0.797685000	-0.343716000	4.150967000
C	-1.163511000	1.843074000	0.826510000
C	-1.644307000	3.205274000	0.955021000
C	-2.743746000	3.348918000	0.064189000
C	-2.927201000	2.072401000	-0.598260000
C	-1.290586000	4.241322000	1.814534000
H	-0.470672000	4.121134000	2.513458000
C	-2.016216000	5.419656000	1.750277000
H	-1.751679000	6.247265000	2.399463000
C	-3.098726000	5.561295000	0.869601000
H	-3.648877000	6.495752000	0.853622000
C	-3.483791000	4.527086000	0.032393000
H	-4.334999000	4.624015000	-0.631699000
C	4.536574000	-1.209609000	-1.911408000

C	5.440203000	-0.205329000	-2.347728000
H	5.218264000	0.565274000	-3.072755000
C	6.673727000	-0.361946000	-1.655736000
H	7.555909000	0.249204000	-1.784504000
C	6.533669000	-1.465599000	-0.768402000
H	7.289113000	-1.847074000	-0.095961000
C	5.212557000	-1.969651000	-0.916529000
H	4.780659000	-2.790551000	-0.359576000
C	3.600001000	1.122171000	0.565263000
H	2.600557000	1.120810000	0.143685000
C	4.657360000	1.994926000	0.218340000
H	4.596745000	2.806302000	-0.494170000
C	5.823807000	1.591757000	0.919368000
H	6.797054000	2.058119000	0.856574000
C	5.486330000	0.453363000	1.706087000
H	6.153840000	-0.089617000	2.360184000
C	4.108980000	0.164757000	1.478376000
H	3.544419000	-0.647618000	1.915151000
B	-1.716335000	-0.107101000	-0.643083000
C	0.363354000	-0.053905000	-1.919259000
O	-0.915922000	-0.334671000	-1.876662000
O	0.988000000	0.431360000	-0.994735000
C	1.003157000	-0.390672000	-3.247380000
C	2.519821000	-0.296970000	-3.187229000
C	3.126392000	-1.425562000	-2.355661000
H	2.511035000	-1.604606000	-1.468460000
H	3.106898000	-2.360899000	-2.930467000
H	2.933112000	-0.313636000	-4.200256000
H	2.778296000	0.669307000	-2.742950000
H	0.658811000	-1.382552000	-3.557355000
H	0.595922000	0.306860000	-3.987257000

3.5. Optimizedcation coordinates of FcCO(CH$_2$)$_2$CO$_2$BSubPc(H)$_{12}$, **11**:

Fe	4.589882000	0.013034000	-0.048853000
N	-3.080022000	-0.046051000	-0.899637000
N	-3.123079000	-2.381370000	-0.562184000
N	-1.245343000	-1.111858000	0.128159000
N	-0.033349000	0.104155000	1.758165000
N	-1.477675000	1.233117000	0.263535000
N	-3.573752000	2.182553000	-0.298840000
C	-3.933823000	1.011283000	-0.820877000
C	-5.249601000	0.467133000	-1.102536000
C	-5.110010000	-0.946203000	-1.184960000
C	-3.710281000	-1.252049000	-0.952433000
C	-6.502826000	1.063064000	-1.192537000
H	-6.609231000	2.138553000	-1.109802000
C	-7.600876000	0.241562000	-1.394768000
H	-8.587859000	0.681512000	-1.486059000
C	-7.463320000	-1.150952000	-1.476110000
H	-8.346567000	-1.761349000	-1.628831000
C	-6.223985000	-1.760461000	-1.357384000

H	-6.117835000	-2.838282000	-1.400551000
C	-1.942906000	-2.282573000	0.050537000
C	-1.313407000	-3.170546000	1.005694000
C	-0.360093000	-2.401248000	1.731430000
C	-0.412960000	-1.050976000	1.206867000
C	-1.568424000	-4.495518000	1.349063000
H	-2.311991000	-5.069280000	0.807911000
C	-0.849174000	-5.049086000	2.394605000
H	-1.018861000	-6.083388000	2.672820000
C	0.086383000	-4.291126000	3.115149000
H	0.618539000	-4.753854000	3.939180000
C	0.327810000	-2.963335000	2.804981000
H	1.026228000	-2.371130000	3.385729000
C	-0.632855000	1.217557000	1.334039000
C	-0.835597000	2.486116000	2.005664000
C	-1.931662000	3.129356000	1.364697000
C	-2.390181000	2.246537000	0.311444000
C	-0.252614000	3.049176000	3.138292000
H	0.558761000	2.544742000	3.650912000
C	-0.743391000	4.261583000	3.593682000
H	-0.297715000	4.725864000	4.466704000
C	-1.820445000	4.896680000	2.957338000
H	-2.183998000	5.840559000	3.348361000
C	-2.434832000	4.332303000	1.852061000
H	-3.284631000	4.805951000	1.374090000
C	4.178187000	0.868704000	-1.914369000
C	3.899117000	1.836052000	-0.915709000
H	2.918217000	2.238647000	-0.704141000
C	5.078521000	2.052989000	-0.166778000
H	5.169549000	2.704034000	0.691928000
C	6.108232000	1.233268000	-0.709163000
H	7.130057000	1.181670000	-0.360197000
C	5.550363000	0.492739000	-1.788024000
H	6.054929000	-0.228857000	-2.414982000
C	3.242841000	-0.678701000	1.427014000
H	2.393580000	-0.113939000	1.795412000
C	4.557165000	-0.682326000	1.950711000
H	4.902669000	-0.110469000	2.801378000
C	5.363734000	-1.508604000	1.125588000
H	6.421109000	-1.694178000	1.254556000
C	4.540021000	-2.022974000	0.083000000
H	4.852337000	-2.671977000	-0.722735000
C	3.228600000	-1.502550000	0.272411000
H	2.379056000	-1.649298000	-0.378249000
B	-1.622734000	0.084586000	-0.666260000
C	0.345110000	0.406841000	-2.093288000
O	-0.945325000	0.226574000	-1.982897000
O	1.120266000	0.467153000	-1.153009000
C	0.818887000	0.520744000	-3.520452000
C	2.214540000	1.124352000	-3.576130000
C	3.231438000	0.239751000	-2.895590000
H	2.534910000	1.211350000	-4.620991000

H	2.222462000	2.130296000	-3.151097000
H	0.826014000	-0.487569000	-3.948938000
H	0.100672000	1.103399000	-4.100548000
O	3.354151000	-0.932477000	-3.159559000

4. References

1. Blom, N.F.; Neuse, E.W.; Thomas, H.G. Electrochemical characterization of some ferrocenylcarboxylic acids. *Transit. Met. Chem.* **1987**, *12*, 301–306.
2. Swarts, P.J.; Conradie, J. Solvent and substituent effect on electrochemistry of ferrocenylcarboxylic acids. *J. Electroanal. Chem.* **2020**, *866*, 114164.
3. Swarts, P.J.; Conradie, J. Electrochemical behaviour of chloro- and hydroxy-subphthalocyanines. *Electrochim. Acta* **2020**, *329*, 135165.
4. Davis, W.L.; Shago, R.F.; Langner, E.H.G.; Swarts, J.C. Synthesis and electrochemical properties of a series of ferrocene-containing alcohols. *Polyhedron* **2005**, *24*, 1611–1616.

© 2020 by the authors. Submitted for possible open access publication under the terms and conditions of the Creative Commons Attribution (CC BY) license (http://creativecommons.org/licenses/by/4.0/).
